# Anti-VEGF therapy selects for clones resistant to glucose starvation in ovarian cancer xenografts

**DOI:** 10.1186/s13046-023-02779-x

**Published:** 2023-08-07

**Authors:** Daniele Boso, Martina Tognon, Matteo Curtarello, Sonia Minuzzo, Ilaria Piga, Valentina Brillo, Elisabetta Lazzarini, Jessica Carlet, Ludovica Marra, Chiara Trento, Andrea Rasola, Ionica Masgras, Leonardo Caporali, Fabio Del Ben, Giulia Brisotto, Matteo Turetta, Roberta Pastorelli, Laura Brunelli, Filippo Navaglia, Giovanni Esposito, Angela Grassi, Stefano Indraccolo

**Affiliations:** 1 Basic and Translational Oncology Unit, Veneto Institute of Oncology IOV-IRCCS, via Gattamelata 64, 35128 Padova, Italy; 2 Immunology and Molecular Oncology Unit, Veneto Institute of Oncology IOV-IRCCS, Padova, Italy; 3 Department of Surgery, Oncology and Gastroenterology, University of Padova, via Giustiniani 2, Padova, 35124 Italy; 4 Department of Biology, University of Padova, Padova, Italy; 5 Medical Oncology 2, Veneto Institute of Oncology IOV-IRCCS, Padova, Italy; 6 Department of Biomedical Sciences, University of Padova, Padova, Italy; 7 Institute of Neuroscience, National Research Council, Padova, Italy; 8 Department of Biomedical and Neuromotor Sciences - DIBINEM, University of Bologna, Bologna, Italy; 9 Immunopathology and Cancer Biomarkers, Centro di Riferimento Oncologico di Aviano (CRO)-IRCCS, Aviano, Italy; 10 Laboratory of Mass Spectrometry, Department of Environmental Health Sciences, Istituto di Ricerche Farmacologiche Mario Negri IRCCS, Milan, Italy; 11 Laboratory Medicine, Department of Medicine-DIMED, University Hospital of Padova, Padova, Italy

**Keywords:** Ovarian cancer, Anti-angiogenic therapy, Glucose deprivation resistance, Mitochondria, Oxidative phosphorylation

## Abstract

**Background:**

Genetic and metabolic heterogeneity are well-known features of cancer and tumors can be viewed as an evolving mix of subclonal populations, subjected to selection driven by microenvironmental pressures or drug treatment. In previous studies, anti-VEGF therapy was found to elicit rewiring of tumor metabolism, causing marked alterations in glucose, lactate ad ATP levels in tumors. The aim of this study was to evaluate whether differences in the sensitivity to glucose starvation existed at the clonal level in ovarian cancer cells and to investigate the effects induced by anti-VEGF therapy on this phenotype by multi-omics analysis.

**Methods:**

Clonal populations, obtained from both ovarian cancer cell lines (IGROV-1 and SKOV3) and tumor xenografts upon glucose deprivation, were defined as glucose deprivation resistant (GDR) or glucose deprivation sensitive (GDS) clones based on their in vitro behaviour. GDR and GDS clones were characterized using a multi-omics approach, including genetic, transcriptomic and metabolic analysis, and tested for their tumorigenic potential and reaction to anti-angiogenic therapy.

**Results:**

Two clonal populations, GDR and GDS, with strikingly different viability following in vitro glucose starvation, were identified in ovarian cancer cell lines. GDR clones survived and overcame glucose starvation-induced stress by enhancing mitochondrial oxidative phosphorylation (OXPHOS) and both pyruvate and lipids uptake, whereas GDS clones were less able to adapt and died. Treatment of ovarian cancer xenografts with the anti-VEGF drug bevacizumab positively selected for GDR clones that disclosed increased tumorigenic properties in NOD/SCID mice. Remarkably, GDR clones were more sensitive than GDS clones to the mitochondrial respiratory chain complex I inhibitor metformin, thus suggesting a potential therapeutic strategy to target the OXPHOS-metabolic dependency of this subpopulation.

**Conclusion:**

A glucose-deprivation resistant population of ovarian cancer cells showing druggable OXPHOS-dependent metabolic traits is enriched in experimental tumors treated by anti-VEGF therapy.

**Supplementary Information:**

The online version contains supplementary material available at 10.1186/s13046-023-02779-x.

## Background

Extensive genetic and phenotypic variation exists among tumors (inter-tumor heterogeneity) but also within tumors (intra-tumor heterogeneity). This diversity can in part be ascribed to the genetic instability that arises through various routes and can influence tumor evolution and patient outcome. Therefore, cancer is often composed of distinct subclonal populations that expand, evolve and undergo selection, continually adapting to the surrounding microenvironment [1, 2]. The subclonal architecture of cancer is dynamic and it can vary during the disease course. Besides the cancer ecosystem, another type of selection is operated by cancer therapeutics: drugs or radiation may destroy cancer cells but also inadvertently provide a potent selective pressure for the expansion of resistant variants [1]. Anti-angiogenic therapy, as for example the anti-VEGF monoclonal antibody bevacizumab, has been approved for treatment of cancer in specific clinical settings [3]. In the case of ovarian cancer, bevacizumab has been approved in clinical practice together with chemotherapy based on results from randomized clinical trials showing significant benefits in terms of progression-free survival, with acceptable tolerability and no detrimental effects on quality of life [4]. Importantly, it has been demonstrated that inhibition of tumor angiogenesis in mouse models generates a selective pressure, driving surviving cancer cells to undergo metabolic reprogramming in order to adapt to the new hypoxic environment and eventually become resistant to therapy [5]. Along this line, our laboratory previously unvealed that bevacizumab caused glucose and ATP deprivation in experimental tumors, leading to AMP-activated protein kinase (AMPK) activation [6]. Glycolysis is generally fostered by anti-angiogenic therapy as the result of a long-term adaptive process [7], but different tumor areas can disclose prevalently glycolytic or oxidative metabolism, as reported by several studies [8–10]. These findings suggest that the harsh hypoxic and glucose-restricted microenvironment of tumors treated with anti-angiogenic therapy might select specific subpopulations of tumor cells endowed with distinctive capability to resist glucose starvation. The concept that glucose concentrations are often decreased in solid tumors compared with the surrounding normal tissue is well-established and prompted investigation of the strategies adopted by cancer cells to maintain their metabolic requirements [11–15]. Previous studies have shown that among cancer cell lines marked differences can be found in their capability to survive glucose limitation, and an RNA interference screen pinpointed mitochondrial oxidative phosphorylation (OXPHOS) as the major pathway required for optimal proliferation under low glucose conditions [16]. Among many metabolic pathways, lipid metabolism is increased in rapidly proliferating cells, because of their tremendous need of energy, membrane biosynthesis and generation of signaling molecules [17, 18]. In cancer cells, fatty acids (FAs) are available from either exogenous sources or from *de novo* FAs synthesis.

In this study, we evaluated whether differences in the sensitivity to glucose starvation existed at the clonal level in ovarian cancer cell lines, investigated the effect of anti-VEGF therapy on this phenotype in patient-derived xenografts and explored the basis of this phenomenon by multi-omics analysis. Moreover, we found significant increased OXPHOS dependent metabolic activities in GDR clones compared with GDS clones and we observed that anti-VEGF therapy caused imbalance of the GDR/GDS ratio in experimental tumors, with substantial enrichment of GDR clones.

## Methods

### Cell culture and treatments

Established ovarian cancer cell lines, including IGROV-1, OC316, SKOV3, A2780, OAW42 and A2774 were used in this study. IGROV-1 cells were purchased from ATCC (Manassas, VA). S. Ferrini (IST, Genoa, Italy) supplied OC316 cells. S. Canevari (INT, Milan, Italy) kindly provided SKOV3, A2780, OAW42 and A2774 cells. Authentication of specific genetic fingerprint by short tandem repeat (STR) DNA profile analysis (PowerPLex 16 HS System, Promega Corporation, Madison, USA) showed that the cell lines presented exactly the same expected *loci* number profile, and confirmed their genetic identity (Suppl. Table 1). IGROV-1, OC316 and SKOV3 cells were grown in RPMI1640 (Euroclone, Pero, Italy) supplemented with 10% fetal bovine serum (FBS) (Life Technologies, Gaithersburg, MD), 1% HEPES (10mM, Cambrex Bioscience, Verviers, Belgium), 1% L-Glutamine (2mM), 1% Sodium Pyruvate (1mM) and 1% antibiotics-antimycotic mix (Gibco-BRL, Grand Island, NY). Cultures were maintained at 37 °C in a humidified 5% CO_2_/ 95% air atmosphere. For glucose deprivation assays, RPMI-1640 medium with L-Glutamine, without glucose and sodium bicarbonate (SigmaAldrich, St. Louis, MO) was used. This medium was supplemented with 10% fetal bovine serum and, when required, 2 mg/ml glucose. The final glucose concentration in glucose-low medium was approximatively 0.2 mM whereas in glucose-high medium it was 11 mM. In some experiments, metformin was used as a mild respiratory chain Complex I inhibitor (MW: 165.62 𝑔/𝑚𝑜𝑙, Sigma-Aldrich, St.Louis, Missouri, US) and oleic acid-albumin from bovine serum was employed (OA, Sigma-Aldrich, St.Louis, Missouri, US, 300 mM).

### Clones generation

Clonal populations were obtained from ovarian cancer cell lines including IGROV-1, OC316, SKOV3, A2780, OAW42 and A2774. In order to obtain single-cell clones, we used the limited dilution method: by dispensing 100 µL per well of a suspension at a concentration of 100 cells/9.6 mL, we obtained, according to Poisson distribution, at most 2 cells per well in around 91% of wells. Specifically, we obtained 35% of empty wells, 37% containing a single cell, and 19% containing two cells. The wells containing more than 2 cells were around 9%. By dispensing instead 50 µL per well of the same suspension, we obtained at most 2 cells in around 98% of wells. Specifically, we obtained 59% of empty wells, 31% of single-cell, and 8% of doublets. The wells containing more than 2 cells were only 2%. Once the clones covered > 80% of the well, they were detached, split into two wells of a new 96-well plate, which were cultured either in standard (11 mM) or low (0.2 mM) glucose medium. Through daily observations, we evaluated by microscopy the behaviour of clones under glucose starvation and after 72 h we classified clones in two categories by evaluating the percentage of confluency with Fiji cell quantification tool (National Institutes of Health, USA). GDS clones were characterised by a percentage of cell confluency below 20%, whereas GDR clones presented a cell confluency above 20% after 72 h under glucose starvation.

### In vivo experiments

For tumor establishment, 8-week-old NOD/SCID mice (Charles River, Wilmington, MA) were subcutaneously (s.c.) injected into both flanks with 0.3–0.5 × 10^6^ tumor cells mixed at 4 °C with liquid Matrigel (Becton-Dickinson; Franklin Lakes, NY). Tumor volume (mm^3^) was calculated as previously reported [19]. When tumors were about 150 mm^3^, anti-human VEGF mAb (bevacizumab) was administered intraperitoneally (i.p.) at 100 µg/dose bi- or tri-weekly to NOD/SCID and mice were sacrificed 48 h after the last treatment. Control mice received i.p. injections of PBS. To obtain GDS and GDR clones from xenografts, we isolated the ex vivo cell cultures from both bevacizumab-treated and control xenografts and calculated the percentage of GDS and GDR clones on the total number of clones that we obtained. The human patient-derived xenograft (PDX) model of ovarian cancer was reviewed and approved by the Veneto Institute of Oncology (IOV) Institutional Review Board and Ethics Committee (EM 23/2017) and was performed in accordance with the Declaration of Helsinki. The patient provided written informed consent to participate in this study. Patient-derived xenograft was previously derived in our lab from cancer cells contained in ascitic effusions from patient bearing epithelial ovarian cancer (EOC) [7, 20], and utilized in this study. PDX was obtained by intraperitoneally injecting 1 × 10^6^ PDOVCA62 cancer cells in NOD/SCID mice. Two days after injection, mice were treated twice per week with i.p. injections of bevacizumab or PBS (control mice) until ethical endopoint.

### Histology and Immunohistochemistry

Five-micron-thick formalin-fixed, paraffin-embedded (FFPE) tumor samples were stained either with hematoxylin and eosin (H&E) or processed for immunohistochemistry (IHC). Staining was performed by using an automatic stainer (LEICA BOND III) using antibodies against cluster of differentiation 31 (CD31) (SZ31, DIANOVA, dilution 1:20), phospho-Histone H3 (pHH3) (#4499 Cell Signaling Technology Danvers, Massachusetts, USA, dilution 1:100) and cleaved Caspase-3 (#9661, Cell Signaling Technology Danvers, dilution 1:50). For image acquisition and analysis, tumor representation and quality of staining were initially evaluated by the pathologist. Slides images were digitally acquired at 20x magnification by Aperio CS2 (Leica Biosystems) and the evaluation of immunochemistry score was assessed through Scanscope Image Analysis software (ImageScope v12.4.0.708). Each marker was analysed by using Aperio membrane Algorithm v9 and microvessel analysis v1 (CD31). Aperio Genie Classifier was trained to recognize tumour tissue, stroma and background (glass) and then combined with Aperio Membrane v9 and Aperio Cytoplasmic v9. Results provided the percentage of cells with different expression of proteins classified in 3+ (highly positive), 2+ (intermediate positive), 1+ (low positive) and 0 (negative).

### Cell viability

Cell viability of clones in glucose deprivation assay in vitro and in PDX-derived ex vivo cultures was evaluated using Annexin V/PI Staining Kit (Roche Applied Sciences; Penzberg, Germany). Cells were stained with 2 µl Annexin-V Fluos, 2 µl Propidium Iodide and 100 µl Hepes buffer, according to the manufacturer’s instruction. Following an incubation of 15 min in the dark, staining was blocked with 200 µl Hepes buffer. Labelled cells were analysed by LSR II cytofluorimeter (Becton Dickinson). Cell viability of GDS and GDR clones in vitro and GDR clones cultured in FBS deprived medium or oleic acid enriched one was calculated by using CellTiter 96® Aqueous One Solution Cell Proliferation Assay (MTS) following manufacturer’s instructions (Promega, Milan, Italy). For pyruvate deprivation experiments, GDS and GDR clones were cultured for 72 h under pyruvate deprivation and cell viability was measured by using Sulforhodamine B Assay Kit (SRB) following manufacturer’s instructions (Abcam, Cambridge, UK).

### Seahorse analysis

Seahorse analysis allows dynamic measure of the oxygen consumption rate (OCR) and extracellular acidification rate (ECAR). We applied this technique to investigate glycolysis and OXPHOS in GDR and GDS clones. To this end, 2.5 × 10^4^ cells per well (n = 5 replicates per sample) were plated in RPMI medium supplemented with 10% FBS. The following day, cells were placed in a running DMEM medium and pre-incubated for 30 min at 37 °C in atmospheric CO2 before starting Seahorse measurements. The cartridge of the instrument was loaded with and dispensed four different metabolic inhibitors at 20 min intervals: oligomycin, carbonyl cyanide 4-(trifluoromethoxy)phenylhydrazone (FCCP), antimycin A and rotenone. Spare respiratory capacity was calculated by subtracting the mean basal respiration values from the maximum respiration values. Glycolytic capacity was calculated as the difference between the mean ECAR following the injection of oligomycin and the basal ECAR values.

### Glucose and lactate measurements

GDS and GDR clones were cultured in 6-well plates under normal conditions and after 72 h supernatants were collected. Glucose and lactate concentrations were determined by colorimetric methods on an automated analyzer (Dimension RxL, Dade Behring, Milan, Italy). Values were normalized to cells number at the end of the incubation period.

### Single-cell acidification assay

Single-cell acidification assay was performed to evaluate the secretion of acid from individual living tumor cells, as previously described [21]. Cells from IGROV-1 clones were splitted into small (picoliter/nanoliter) aqueous droplets in oil (making a water-in-oil emulsion) using microfluidic technology. Each droplet contains at most a single cell and molecules secreted by this single cell are retained by the droplet. To screen droplets with higher throughput in a semi-automated way, we engineered an inverted microscope, so that each droplet can be analysed using laser-induced fluorescence at approximately 1 kHz. For each droplet the ratio of emitted fluorescence intensities at 580 and 630 nm is calculated in real time.

### Mass spectrometry-based untargeted metabolomics

GDR and GDS cells clones were grown for 48 h in biological triplicate. At 48 h each clone was rapidly rinsed in saline solution (~ 2s), aspirated, and metabolism was quenched by adding ~ 15mL of liquid N2 to the dish. The plates were then stored at -80 °C, and extracted and analysed within seven days. Extraction was done by adding 1 mL of ice-cold 90% 9:1 MeOH:CHCl3 to each plate and cells were scraped centrifuge at 4 °C for 15 min at 10000xg. Supernatants were collected for metabolomics analysis. Flow Injection Analysis High resolution mass spectrometry (FIA-HRMS) was used for untargeted metabolomics [22]. A portion of the extract (8 µL) was analysed by LTQ-Orbitrap XL Mass Spectrometer (Thermo Fisher Scientific) equipped with an electrospray source operated in negative and positive modes. Each run was carried out by injecting 8 µL of sample extract at a flow rate of 50 µL/min (Agilent 1200 Series) of mobile phase consisting of isopropanol/water (60:40, v/v) buffered with 5 mM ammonium at pH 9 for negative mode and methanol/water (60:40, v/v) with 0.1% formic acid at pH 3 for positive mode. Reference masses for internal calibration were used in continuous infusion during the analysis (m/z 210.1285 for positive and m/z 212.0750 for negative ionization). Mass spectra were recorded from m/z 50 to 1 000 with 60 000 resolutions. Source temperature was set to 240 °C with 25 L/min drying gas and a nebulizer pressure of 35 psig. MS/MS fragmentation pattern of the significant features was collected and used to confirm metabolite identity. All data processing and analysis were done with Matlab R2016a (The Mathworks, Natick, MA) using an in-house developed script [23]. The statistical significance of single metabolite between GDR vs. GDS clones were computed by univariate pairwise comparison Mann–Whitney–Wilcoxon test (JMP pro12, SAS). For biological interpretation of the metabolomic data, we mapped the significant metabolites into biochemical network using MetaboAnalyst (www.metaboanalyst.ca). Enrichment analysis (EA) tools were used to identify metabolic pathways most likely to be involved in GDS, GDR differences.

### Whole exome sequencing (WES) analysis

WES was performed on IGROV-1 (3 GDR and 3 GDS) and SKOV3 clones (3 GDS and 3 GDR). Genomic DNA was extracted with Easy DNA kit (Life Technologies), quantified with Qubit 2.0 Fluorometer (Invitrogen, Carlsbad, CA) and subjected to quality control using agarose gel prior to enzymatic DNA fragmentation. SKOV3 samples were sequenced using the SureSelectXT Low Input Human All Exon V7 48.2 Mb kit (Agilent, Santa Clara, CA), whereas IGROV-1 samples were sequenced by All Exon V6, on NextSeq 500 (Illumina, San Diego, CA) in paired-end mode (2 × 100 bp). Sequencing reads were then analysed with the Alissa Align & Call Software (version 1.0, Agilent Technologies, Santa Clara, CA). Specifically, reads were trimmed and aligned to the human genomic reference hg19 (GRCh37) with default parameters. After reads de-duplication, aligned reads were used for somatic variant calling with Alissa SNPPET according to default threshold values. Single Nucleotide Variant (SNV) annotation and selection was performed using the Alissa Interpret module (version 1.0, Agilent Technologies, Santa Clara, CA). Briefly, exonic SNVs indicated as confident calls (PASS) were considered for further investigation and filtered considering a variant allele frequency cut-off value of 20%, excluding common polymorphisms (MAF > 1% in major population databases), synonymous and intronic SNVs (30,31).

### Mitochondrial DNA sequencing

The mitochondrial genome sequencing was carried out using an NGS approach [24]. Briefly, the entire mtDNA molecule was amplified with two long PCR amplicons (9.1 kb and 11.2 kb), the library was constructed by Nextera XT DNA Library Preparation Kit (Illumina, San Diego, CA) and sequenced on MiSeq System (Illumina, San Diego, CA), using the 300-cycle reagent kit. Reads were aligned to mtDNA reference sequence (NC_012920.1). Population frequencies and annotation of SNVs (single nucleotide variant) were recovered from public database Mitomap (http://www.mitomap.org).

### Transcriptome analysis

Microarray experiments were performed in both IGROV-1 and SKOV3 cell lines. For each cell line, a total of 30 samples, corresponding to 10 clones (5 GDR, 5 GDS) at basal (0 h) and at two time points of glucose deprivation (6 h, 24 h), were collected. RNA quality and purity control was assessed with the Agilent Bioanalyzer 2100 (Agilent Technologies, Waldbronn, Germany) and Eukaryote total RNA Nano Assay (Agilent). For microarray expression experiments, only total RNA of high quality was used (RIN > 7). RNA samples that passed the high quality controls were diluted to 100 ng in a total volume of 3 µl DEPC treated water. In vitro transcription and biotin labelling were performed according to GeneChip 3’IVT Express kit protocol (Affymetrix, Santa Clara, CA). Following fragmentation, biotinylated cRNA was hybridized for 16 h at 45 °C to GeneChip™ PrimeView™ Human Gene Expression Arrays in an Affymetrix GeneChip Hybridization Oven 645. Affymetrix Fluidics Station 450 was used to stain and wash the chips. Arrays were then scanned on Affymetrix GeneChip Scanner GCS3000 and the image (*.DAT) files were processed using the Affymetrix GeneChip Command Console (AGCC) software v.5.0 to generate cell intensity (*.CEL) files. Prior to transcriptional data analysis, chip quality assessment was executed using the Affymetrix Expression Console software v.1.4 and for every array all quality metrics were found within boundaries. Bioinformatic analysis was carried out in the R statistical environment using Bioconductor packages [25]. Data were preprocessed using the RMA algorithm [26]. Given the specific design of the experiments, thought to obtain comparisons both between independent groups of clones (GDR vs. GDS comparisons at fixed time point) and between paired clones (over time comparisons for GDR or GDS clones), the clone variable was treated as a random effect [27]. Differential expression analysis was performed using the limma package, by linear modelling, moderating the t-statistics by empirical Bayes shrinkage [28]. To correct for multiple testing, the Benjamini and Hochberg’s method was applied. Gene Set Enrichment Analysis (GSEA) was performed to evaluate the functional significance of curated sets of genes. Genes were ranked by moderated t-statistics and fast pre-ranked GSEA, as implemented in [29], was run against the KEGG and Reactome canonical pathways present in the “c2.cp.kegg” and “c2.cp.reactome” sub-collections of the Molecular Signatures Database. Gene sets with a Benjamini-Hochberg adjusted p-value < 0.10 were considered significantly enriched.

### Western blotting

The proteic lysates were derived from cells previously cultured in a 6-well plate for 24 h under normal conditions and under glucose starvation. For the lysis we used a Lysis Buffer (NP-40 1%, NaCl 150 mM, Tris HCl pH 7.5 50 mM, EDTA 2 mM, NaF, Na3VO4 and protease inhibitor cocktail), in which the cells were resuspended. About 30 µ𝑔 of proteins were separated in a midi polyacrylamide gel 4–12% (Life Technologies) transferred on a nitrocellulose membrane (GE Health Care, Glattburg, Switzerland). After the transfer, the protein of interest was identified by staining the membrane overnight with the following primary antibodies:

- Mouse anti-α-TUBULIN, 1:4000 (Sigma-Aldrich);
- Rabbit anti-MCT1, 1:1000 (EMD Millipore, AB3538P);
- Rabbit anti-MCT4, 1:300 (Santa Cruz Biotechnology. Sc-50,329);
- Rabbit anti-CD36, 1:1000 (Novus Bio, NB400-144);
- Rabbit anti-FAS, 1:1000 (Cell Signalling Technology, #3180);
- Rabbit anti-CPT1A, 1:1000 (Cell Signalling Technology, #12,252);
- Rabbit anti-pACC, 1:1000 (Cell Signalling Technology, #3661).

The day after, membrane was washed and incubated for 60 min with a horseradish conjugated secondary antibody (goat anti-rabbit, IgG-HRP, 1:5000, Santa Cruz Biotechnology; horse anti-mouse, IgG-HRP, 1:5000, Cell Signalling Technology). The detection was attained using Western Lightning plus ECL reagents (PerkinElmer) and the signal for each of the proteins of interest was then acquired using UVITEC Alliance (Cambridge, UK), and standardized on the signal of a housekeeping protein (Tubulin or Actin).

### BODIPY staining

Cells were stained with 2 µM BODIPY 493/503 (Thermo Fisher Scientific, Waltham, MA, USA) for 15 min at 37 °C and then were analysed using LSRII flow cytometer (BD Biosciences, San Jose, CA, USA).

### Reverse transcription-PCR and quantitative PCR

Total RNA was isolated using RNAeasy® Mini Kit (Qiagen, Venlo, Netherlands) according to manufacturer’s instructions. cDNA was synthesized from 0.3 to 1 µg of total RNA using High Capacity RNA-to-cDNA Kit (Thermo Fisher). Real-time PCRs for SLC16A1 (MCT1) (*Forward* 5’-TGTTGTTGCAAATGGAGTGT-3’, *Reverse* 5’-AAGTCGATAATTGATGCCCATGCCAA-3’) were performed using Platinum® SYBR® Green (Thermo Fisher) in an ABI Prism 7900 HT Sequence Detection System (Thermo Fisher). Results were analysed using the ΔΔCt method with normalization against HMBS gene expression.

### Mitochondrial mass

Mitochondrial mass of GDS and GDR clones under normal conditions or upon glucose deprivation was measured using MitoTracker™ Deep Red FM (Invitrogen, Carlsbad, CA) following manufacturer’s instructions. Verapamil (1µL/mL) was used to block probe leaking.

### Statistical analysis

Results were expressed as mean value ± SD. Statistical comparison between two sets of data was performed using the unpaired Student’s *t* test (two-tailed). Statistical significance is indicated as follows: *P < 0.05; **P < 0.01; ***P < 0.001. For in vivo experiments with PDX, survival was evaluated using the Kaplan–Meier method (log–rank test) with SigmaPlot software.

## Results

### Selection of ovarian cancer cell clones based on their response to glucose limitation

**Fig. 1:**
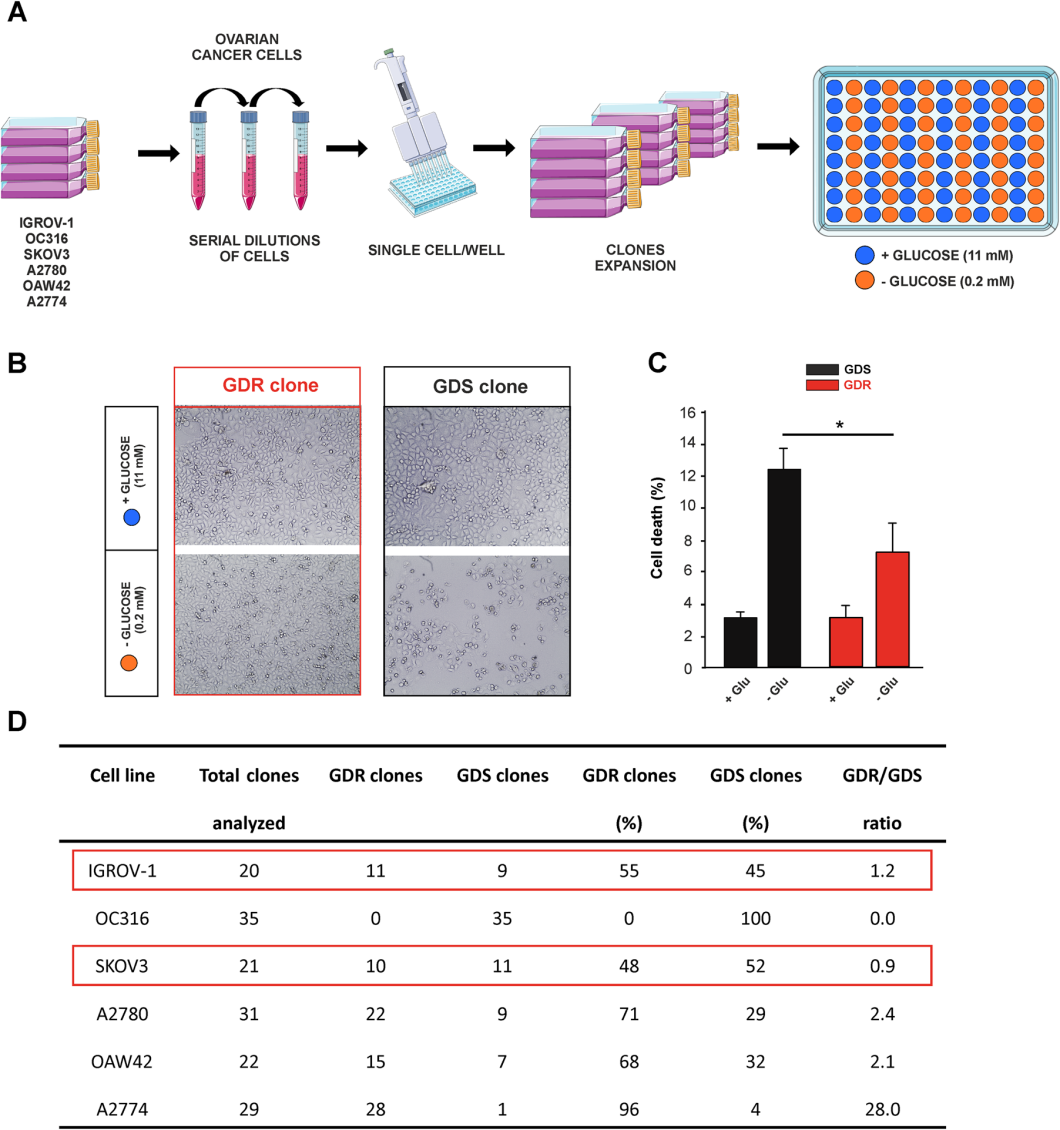
Generation of glucose deprivation resistant (GDR) or sensitive (GDS) clones. **A** Experimental workflow describing the protocol to generate GDR or GDS clones from ovarian cancer cell lines (IGROV-1, OC316, SKOV3, A2780, OAW42 and A2774). Clonal populations of cancer cells were obtained by following a protocol based on serial cell dilutions: 0.5, 1, 2 cells per well were seeded in 96-well plates and plates where clones developed in < 50% of wells were selected for further expansion and cultured either in standard (11mM) or low (0.2mM) glucose medium. **B)** Representative images of SKOV3 clones cultured in standard or low glucose conditions for 72 h and then classified in two arbitrary categories including GDR and GDS clones by microscope observation. **C** SKOV3 GDR and GDS clones were cultured either in standard or low glucose medium and after 72 h cell death was measured by using Annexin V/PI staining. (* *p* < 0.05). **D** Proportions of GDR and GDS clones in ovarian cancer cell lines: total clones analyzed were subjected to glucose limitation regimen (0.2 mM) and percentage (%) of GDR clones and GDS clones were calculated on the GDR/total number of clones ratio and GDS/total number of clones ratio, respectively. The GDR/GDS ratio was calculated as GDR percentage/GDS percentage ratio

To investigate cellular sensitivity to glucose starvation at the clonal level, we obtained populations of clones from several established ovarian cancer cell lines (IGROV-1, OC316, SKOV3, A2780, OAW42 and A2774). Single tumor cells were isolated from each cell line and different clones (range n = 20–35) were derived and expanded in vitro (Fig. 1A). Clones were subjected to glucose limitation regimen and, after 72 h, it was possible to discern two types of clones based on their different morphology and degree of confluency. GDR clones survived under low glucose concentrations (0.2 mM) by conserving their typical adhesive morphology and maintaining a monolayer pattern. On the contrary, GDS clones showed reduced confluency at microscopical observation (Fig. 1B) and disclosed a compromised viability at the same time point (Fig. 1C). The proportion of GDR and GDS clones varied depending on the cancer cell line: A2774 and A2780 cell lines contained an excess (> 80%) of GDR clones, whereas others, such as OC316 were composed entirely by GDS clones (Fig. 1D). IGROV-1 and SKOV3 cell lines were selected for further investigations because of their balanced proportions of GDS and GDR clones (GDR/GDS ratio close to 1) (Fig. 1D). These findings demonstrated an unexpected clonal diversity in ovarian cancer cell lines, with different cell populations that responded oppositely to glucose starvation.

### Anti-VEGF therapy selects GDR clones endowed with a more tumorigenic potential *in vivo*

**Fig. 2:**
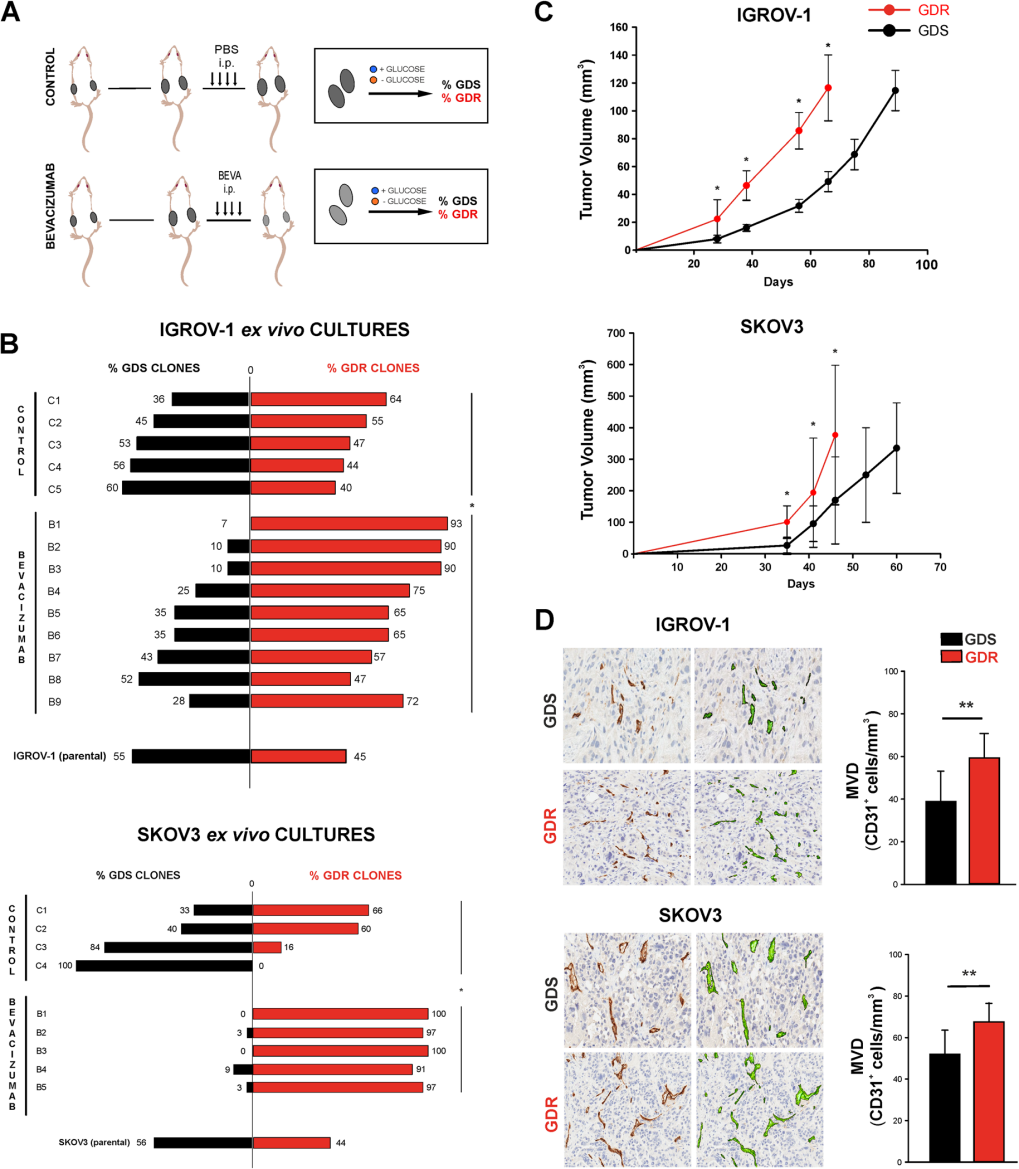
GDR and GDS clonal populations in IGROV-1 and SKOV3 ex vivo cultures and in vivo xenografts.** A** Schematic overview of ovarian cancer xenograft models treated with bevacizumab or PBS (as CONTROL) and glucose deprivation assay in vitro. **B** GDR and GDS clone proportion in IGROV-1 ex vivo control cultures (CONTROL; *n* = 5) and treated cultures (upper panel), (BEVACIZUMAB; *n* = 9). GDR and GDS clone proportion in SKOV-3 ex vivo control cultures (CONTROL; *n* = 4) and treated cultures (lower panel), (BEVACIZUMAB; *n* = 5). The bars represent the percentages of GDR clones and GDS clones, calculated on the GDR/total number of clones ratio and GDS/total number of clones ratio in each ex vivo culture (*p < 0.05). **C** In vivo growth curve of xenografts from GDR and GDS clones of IGROV-1 and SKOV3 cell lines. The growth curves report the mean values ± SD of GDR tumors (*n* = 12) and GDS tumors (*n* = 12) (*p < 0.05) **D** Microvessel density (MVD) of GDR and GDS clones-derived xenografts tissues was quantified by image analysis on digitalized slides. Columns show mean ± SD values (*n* = 5 GDR and 5 GDS clones). On the right panel, representative images of CD31 staining on GDR or GDS tissue slides. The applied green mask represent the visualization of the Aperio microvessels algorithm which was used for digital quantification; Hematoxylin counterstain, original magnification ×20 (**p < 0.01)

Anti-VEGF therapy caused dramatic depletion of glucose and ATP in ovarian cancer xenografts [6]. To investigate whether anti-VEGF therapy perturbed the GDR/GDS ratio in tumor xenograft models, we generated IGROV-1 and SKOV3 tumor xenografts and treated them with bevacizumab (BEVA) for 4 weeks. At sacrifice, we derived ex vivo cell cultures from tumors and performed analysis of GDR and GDS clones (Fig. 2A). The mean percentages of GDR and GDS clones (50% ± 9.56%) in IGROV-1 control tumors were similar to the proportion found in IGROV-1 parental cell line (55% GDR vs. 45% GDS), whereas the average proportion of GDS clones obtained from control SKOV3 tumors (64% ± 32.8% GDS vs. 34% ± 32.5% GDR) was slightly higher than the percentage found in the parental cell line (56% GDR vs. 44% GDS), due to two control tumors (C3, C4) being enriched for GDS clones (Fig. 2B, black bars). A remarkable imbalance was found among GDR and GDS clones in ex vivo cultures from BEVA-treated tumors for IGROV-1 xenografts, with a significant excess of GDR clones (73% ± 15.97%) respect to GDS clones (27% ± 15.7%) in most tumors analyzed and an almost total enrichment for GDR (97% ± 3.6%) respect to GDS (3% ± 3.6%) clones in SKOV3 xenografts (Fig. 2B, Suppl. Table 2). The tumorigenic potential of GDR and GDS derived from control tumors clones was further investigated. The in vivo growth rate of GDR clones was significantly higher compared with GDS clones both in IGROV-1 and SKOV3 xenografts (Fig. 2C). The increased capability of GDR clones to form tumors in vivo was likely not due to intrinsic differences in cell proliferation because similar numbers of pHH3 positive cells were observed in tumors (Suppl. Figure 1 A). Notably, microvessel density (MVD), which is often used as a surrogate for intratumoral angiogenic activity [30], was quantified in GDS and GDR tumor xenografts by IHC. The number of CD31-positive endothelial cells per area unit was higher in GDR-derived tumors when compared with GDS-derived tumors (Fig. 2D). Given the well-established role of angiogenesis in promoting tumor growth, it is tempting to speculate that the increased in vivo growth rate of GDR clones could in part be accounted for by their increased angiogenic capacity. No difference was found in apoptosis between GDR and GDS tumors, whereas SKOV3 tumors were less apoptotic than IGROV-1 ones (Suppl. Figure 1B). Finally, GDS-derived tumors presented larger necrotic areas respect to GDR tumors (Suppl. Figure 1C). These results suggested that anti-VEGF therapy led to marked enrichment of GDR clones that were fit to form tumors under the unfavourable nutrient conditions of the subcutaneous xenograft tumor microenvironment. Finally, we investigated whether therapy-associated enrichment of a glucose deprivation-resistant population could occur in a patient-derived xenograft (PDOVCA62) model of ovarian cancer. Bevacizumab significantly extended the survival of the mice from 29 ± 4 to 47 ± 6 days (mean ± SD) in control and treated groups, respectively (Suppl. Figure 2A). As it was not possible to derive single-cell clones from PDOVCA62 cells, viability of tumor cells from the ascitic fluid sample obtained at sacrifice was analysed under glucose deprivation *in vitro.* An enrichment of GDR cell populations in PDOVCA62 tumors from bevacizumab-treated mice compared with control mice (Suppl. Figure 2B) was observed, in line with results obtained in s.c. tumor xenografts models.

### GDR clones are endowed with an OXPHOS-dependent metabolic phenotype

**Fig. 3:**
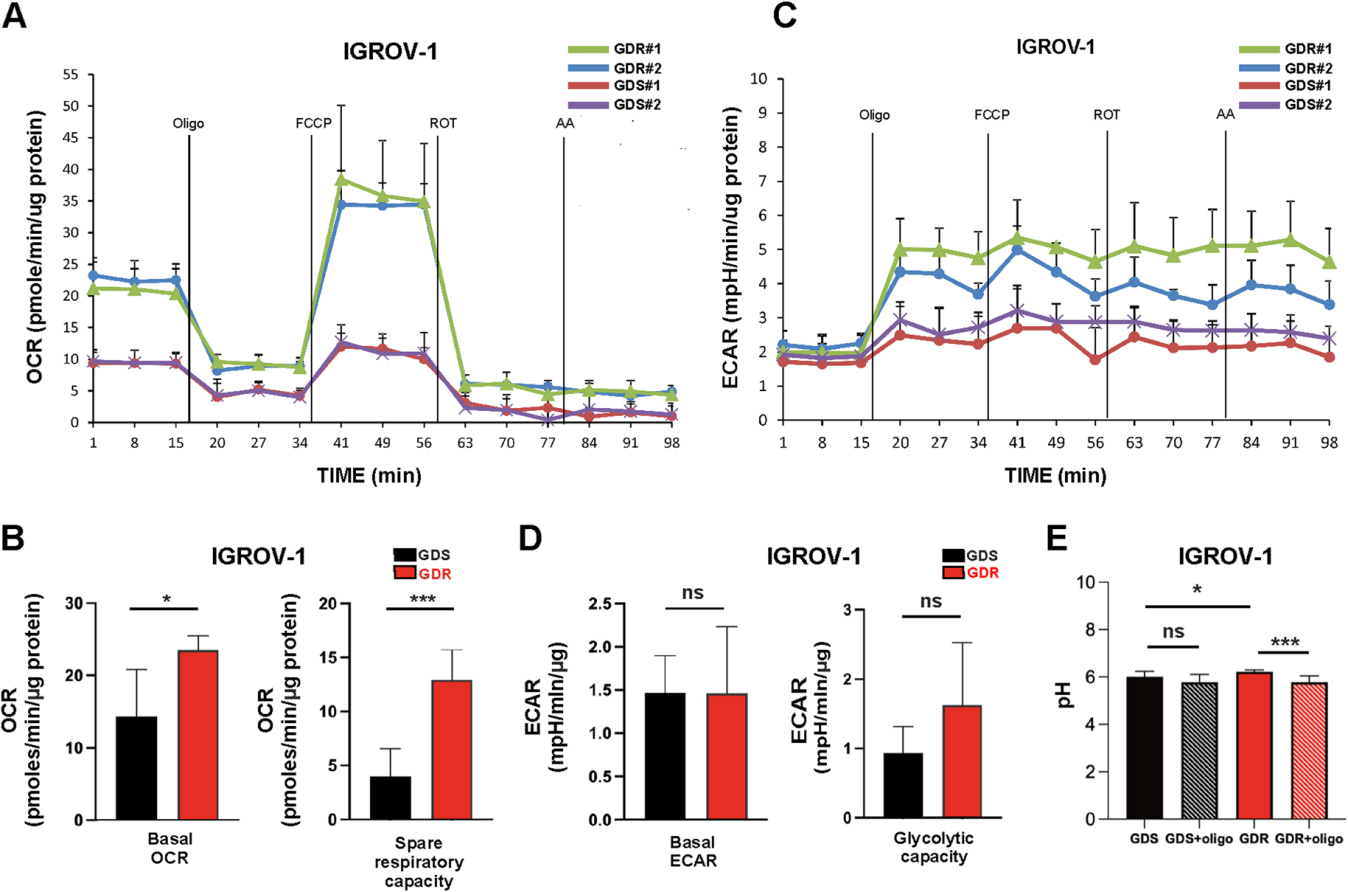
Glycolysis and OXPHOS activity in IGROV-1 GDR and GDS clones. **A** Seahorse measurements of oxygen consumption rate (OCR) of two representative IGROV-1 GDR and GDS clones (GDR#1, GDR #2, GDS #1 and GDS#2). Four different metabolic inhibitors were administered at 20 min intervals: oligomycin (1µM), followed by carbonyl cyanide trifluoromethoxyphenylhydrazone, (FCCP, 0.4 µM), antimycin (1µM) and rotenone (1µM) over 2 h. **B** Basal respiration (left panel) and spare respiratory capacity (right panel) of GDR and GDS clones (4 GDS vs. 6 GDR, 4 GDS vs. 6 GDR, respectively). (*p < 0.05, ***p < 0.001). **C** Seahorse measurements of extracellular acidification rate (ECAR) of the same two representative IGROV-1 GDR and GDS clones (GDR#1, GDR #2, GDS #1 and GDS#2). **D** Basal ECAR (left panel) and maximal glycolytic capacity (right panel) of GDR and GDS clones (4 GDS vs. 6 GDR, 4 GDS vs. 6 GDR, respectively) (ns: not significant). **E** Single-cell metabolism analysis of IGROV-1 GDS and GDR clones. pH values were obtained from the 580/630 nm ratio of SNARF-5 F fluorescent signal. Clones were treated with oligomycin and not treated cells were used as control (ns: not significant, *p < 0.05, ***p < 0.001)

The striking effects of bevacizumab treatment on the GDS/GDR ratio in tumors prompted us to investigate metabolic features associated with the different phenotypes. To functionally investigate metabolic activity, measurements of OXPHOS and glycolysis were obtained in IGROV-1 and SKOV3 clones by using Seahorse technology. At the basal level, IGROV-1 GDR clones were characterized by higher OCR compared with GDS clones whereas SKOV3 GDR clones were similar to GDS clones (Fig. 3A, B; Suppl. Figure 3A, B). With regard to ECAR, no significant differences were found between IGROV-1 GDR and GDS clones, whereas SKOV3 GDS clones were characterized by higher ECAR compared with GDR clones (Fig. 3C, D; Suppl. Figure 3C, D). In response to mitochondrial activity perturbations induced by FCCP, OCR significantly increased in IGROV-1 GDR compared with GDS clones, suggesting that IGROV-1 GDR clones are endowed with increased spare respiratory capacity (Fig. 3B), whereas SKOV3 GDR clones did not respond by increasing OCR (Suppl. Figure 3B). Oligomycin (a mitochondrial inhibitor of ATP synthase) triggered an increase in ECAR in both IGROV-1 and SKOV3 GDR and GDS clones (Fig. 3C, Suppl. Figure 3C) but, interestingly, GDR clones showed higher maximal glycolytic capacity compared to GDS clones (Fig. 3D, Suppl. Figure 3D). Altogether, these observations suggested a metabolic heterogeneity between IGROV-1 and SKOV3 clones at the basal level and, in particular, increased OXPHOS dependent metabolism in IGROV-1 GDR compared with GDS clones, which was not shared by SKOV3 GDR clones. To measure the extent of secreted acid from GDR and GDS clones, we performed single-cell acidification assay by using tumor cells compartmentalized in picoliter droplets [21]. In the presence of glucose at the basal level, IGROV-1 GDR clones showed higher pH values compared to GDS clones, by suggesting a lower acidification rate (Fig. 3E). Oligomycin treatment was able to induce a significant decrease in the pH value in GDR clones when compared to non-treated clones. However, the pH value of GDS clones was not significantly affected by oligomycin treatment (Fig. 3E).

Untargeted metabolic profiling was performed to further investigate the metabolic differences between GDR and GDS clones under basal growth conditions. The major metabolic differences between GDR and GDS IGROV-1 clones were alterations in metabolites abundance belonging to the Warburg effect (28 metabolites) such as glutamate metabolism, ammonia recycling, and urea cycle (Suppl. File 1). In agreement with what previously observed, all the mapped metabolites were involved in biochemical reactions involving the mitochondria. Glucose consumption and lactate production were measured in IGROV-1 and SKOV3 clones. IGROV-1 GDS and GDR clones demonstrated comparable levels of glucose consumption and lactate production (Suppl. Figure 3E) whereas SKOV3 GDS clones showed slightly increased glucose consumption and produced more lactate compared to GDR clones (Suppl. Figure 3 F). Finally, we sought to investigate if the metabolic differences observed in GDS/GDR clones from IGROV-1 and SKOV3 cells existed also in the ovarian cancer cell lines with an almost total enrichment towards GDS (OC316 cell line) or GDR clones (A2774 cell line). As previously reported [6], OC316 cells were endowed with both higher basal ECAR and glycolytic capacity values respect to A2774 cells by indicating a more glycolytic metabolism upon glucose availability (Suppl. Figure 4 A). Upon glucose deprivation, a higher percentage of cell death was observed in OC316 respect to A2774 cells, in line with their GDS phenotype (Suppl. Figure 4B). Pyruvate deprivation induced a significant increase in the percentage of cell death only in GDR-enriched A2774 cell line, but not in the GDS-enriched OC316 cell line, thus suggesting the dependence of A2774 cells from this nutrient, as previously observed for IGROV-1 GDR clones (Suppl. Figure 4 C). Altogether, these findings suggest that certain metabolic traits of GDR/GDS clones can be found across different ovarian cancer cell lines.

### GDR and GDS clones lack peculiar genetic signatures

To investigate possible genetic markers associated with GDS and GDR clones, whole exome sequencing (WES) was carried out on 12 clones including 3 GDR vs. 3 GDS IGROV-1 and SKOV3 clones. WES data were subsequently analysed by searching for shared or exclusive mutations within each group. After variant filtering, exome analysis revealed 425 unique variants with VAF > 20% in IGROV-1 clones. Among them, 52 (12%) were shared by all six samples in both GDR and GDS, whereas 182 (42.8%), 23 (5.4%), 14 (3.3%) additional variants were shared by 5, 4, 3, 2 samples, respectively (Suppl. Figure 5). 33 exclusive variants were carried by clone GDR_11_12, 102 by clone GDR_12_19, 16 by clone GDS_10_12, and 15 by clone GDS_10_25, 15 by clone GDS_10_26 (Suppl. Table 3). No variant was shared by GDR clones, whereas 1 missense variant was shared by all 3 GDS samples and absent in all GDR samples: *MTG1* c.338G > A, p.Cys113Tyr (VAF 32% in GDS10_12; 21,8% in GDS10_26, 22,8% in GDS10_25). The *MTG1* gene encodes for the mitochondrial ribosome associated GTPase 1, involved in the regulation of mitochondrial translation and of respiratory system process (https://www.ncbi.nlm.nih.gov/gene/92170). This variant is predicted to be deleterious and is reported in cosmic database as associated to endometrial carcinoma (COSV100448883), but lacks functional characterization.

With regard to SKOV3 clones, WES analysis showed 201 unique variants: 35 (17.4%) shared by all samples as well as 9 (4.5%), 10 (4.9%), 7 (5.5%), 11 (5.5%) variants were shared by 5, 4, 3, 2 clones, respectively (Suppl. Figure B). Again, most variants were found to be private: 17 were exclusively carried by clone GDR_3–9, 13 by clone GDR_3–8, 31 by clone GDR_3–19, 31 by clone GDS_4–8, and 19 by clone GDS_3–5 (Suppl.Table 4).

One variant was shared by 2 GDS SKOV3 clones, *WASHC2A* c.1706T > C p.Leu569Ser (VAF 27% in GDS_4–8, 23% in GDS_3–5), and one variant was shared by 2 GDR clones, *MFSD4B* c.604del p.Cys202Valfs*4 (VAF 22% in both GDR_3–19 and GDR_3–8).

The *WASHC2A* missense variant has been observed in various cancers as neutral; while the *MFSD4B* variant has been previously described in gastric and colorectal cancer [31][31][31][31][32], and is likely to cause loss of function of the protein, which is predicted to enable glucose transmembrane transporter activity.

By merging IGROV-1 and SKOV3 WES data, we observed a higher number of SNVs carried by SKOV3 clones with respect to IGROV1 clones, possibly due to the different enrichment performance of the two exome panels versions. In any case, we did not detect any shared variants within GDS or GDR groups, except for one variant common to all 12 samples (Suppl. Figure 6), *SPIN2B* c.136 C > T p.Arg46Cys, with an overall VAF from 36.7 to 58.4%.

In a previous study, mitochondrial DNA mutations accounted for limited resistance of cancer cell lines to glucose limitation [16]. Mitochondrial DNA analysis of IGROV-1 GDR and GDS clones showed 32 variants: 28 homoplastic changes shared by all samples, 1 heteroplasmic change shared by all samples, and 3 heteroplasmic changes present in single samples (Suppl. Table 5). Two of them are coding variants, both variants absent in Mitomap database (https://www.mitomap.org/MITOMAP), detected respectively in *MT-ND5* gene of IGROV-1 GDR_7 at an heteroplasmy level of 9% and in *MT-RNR2* gene of IGROV-1 GDS_9 at an heteroplasmy level of 21%. These mutations however were present at low levels and are therefore not likely to affect cell metabolism, thus indicating the lack of a peculiar genetic signature at the mitochondrial level that could have an impact on metabolic features. Altogether, these results indicate that genetic alterations are not likely to account for the marked difference in tolerance to glucose starvation observed in GDR versus GDS clones.

### GDR clones exacerbate an OXPHOS-related transcriptional program to overcome glucose deprivation stressful stimulus

**Fig. 4:**
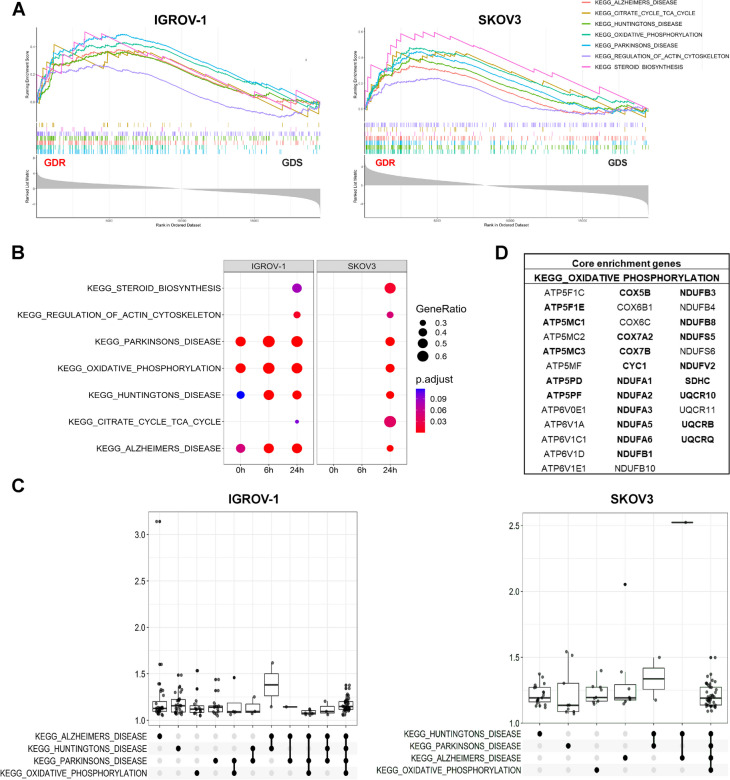
Activation of OXPHOS pathway characterizes GDR clones after 24 h of glucose deprivation. **A** GSEA enrichment plots showing the seven significantly upregulated pathways in GDR vs. GDS clones at 24 h of glucose deprivation in IGROV-1 (left panel) and SKOV 3 (right panel). **B** Dot plot displaying the activation profiles of the shared pathways over time, x-axis represents the time course (hours of glucose deprivation), the adjusted p-value (p.adjust) indicates the significance of each pathway while GeneRatio the number of core enrichment genes/total number of genes in the pathway. **C** UpSet plots for the 4 most upregulated KEGG pathways shared between IGROV-1 and SKOV3 models. This visualization allows discerning core genes that uniquely belong to a pathway or are shared between two or more pathways. On y-axis the fold change distribution of core enrichment genes in the different groups is displayed. **D** List of the 37 genes belonging to the core enrichment of KEGG_OXIDATIVE_PHOSPHORYLATION pathway for both the IGROV-1 and the SKOV 3 model. Genes in bold are in the core enrichment of 4 KEGG pathways: OXIDATIVE PHOSPHORYLATION, ALZHEIMERS DISEASE, HUNTINGTONS DISEASE and PARKINSONS DISEASE

To investigate whether the metabolic differences observed in GDR and GDS clones were associated with distinct signatures at the transcriptional level, a differential expression (DE) analysis of GDR and GDS clones cultivated under either normal-glucose or low-glucose condition was performed. Based on previous studies [32], two time points (6 and 24 h) were selected to investigate both early and late transcriptional effects of glucose deprivation in IGROV-1 and SKOV3 models. Glucose deprivation caused massive modulation of genes in all clones (Suppl. Table 6) and, to characterize its action from a functional point of view, GSEA on KEGG pathways was conducted. The analysis over time, comparing cells at different time points during glucose starvation (6 or 24 h) with the basal condition (0 h), disclosed several altered KEGG pathways in both GDR -glu vs. GDR + glu and GDS -glu vs. GDS + glu comparisons (Suppl. Figure 7 and Suppl. Figure 8, Suppl. File 2). DE analysis between GDR and GDS clones at the same time point did not show differentially expressed genes between the two populations, except for few tens of probes for IGROV-1 at 24 h of glucose deprivation (Suppl. Table 6). However, the GSEA method showed several significantly altered KEGG pathways in GDR with respect to GDS clones (Suppl. File 2). We selected the common perturbed pathways between the two cell models in order to identify the functional processes characterizing GDR clones. Specifically, after 24 h of glucose deprivation, two downregulated pathways (KEGG N-Glycan biosynthesis and KEGG protein export) and, more interestingly, seven upregulated pathways were shared (Fig. 4A). To compare the behaviour of IGROV-1 and SKOV3 GDR clones, the seven upregulated pathways were analyzed over time, highlighting a common modulation pattern of the same gene sets but with different timing (Fig. 4B). Specifically, IGROV-1 GDR clones disclosed a basal (0 h) upregulation of KEGG pathways strictly related to metabolic changes such as OXPHOS, Parkinsons disease, Huntingtons disease, Alzheimers disease, compared to GDS clones, thus confirming their previously demonstrated OXPHOS metabolic propensity. Moreover, after 6 h of glucose deprivation, IGROV-1 GDR clones showed an even enhanced upregulation of OXPHOS-related pathways that was maintained after 24 h. Differently, SKOV3 GDR clones showed significant activation of the same processes of IGROV-1 clones only after 24 h of glucose deprivation and not at the other time points analyzed. To better characterize the common transcriptional response between IGROV-1 and SKOV3 GDR clones, that were able to survive the stressful glucose deprivation stimulus, we selected the four pathways most strongly upregulated in GDR clones of both models (adjusted p < 0.0005) after 24 h of glucose deprivation and visualized the complex networks of interconnections among core enrichment genes (Suppl. Figure 9). Finally, to further explore the pathways crosstalk between the two models, gene expression distribution of core enrichment genes was displayed by UpSet plot (Fig. 4C). Given the high overlap of core genes involved in OXPHOS pathway and the other strongly upregulated KEGG pathways (Parkinsons disease, Huntingtons disease and Alzheimers disease; Suppl. Figure 9, Fig. 4C), the parallel activation of these pathways both in IGROV-1 and SKOV3 models was expected (Fig. 4B). Core genes sustaining the significant activation of OXPHOS pathway in GDR vs. GDS clones are listed in Fig. 4D and represent a promising starting point for the identification of a GDR signature in ovarian cancer.

### SKOV3 GDR clones accumulate and metabolize external lipids, whereas GDS clones rely on accumulation and synthesis of new FAs

**Fig. 5:**
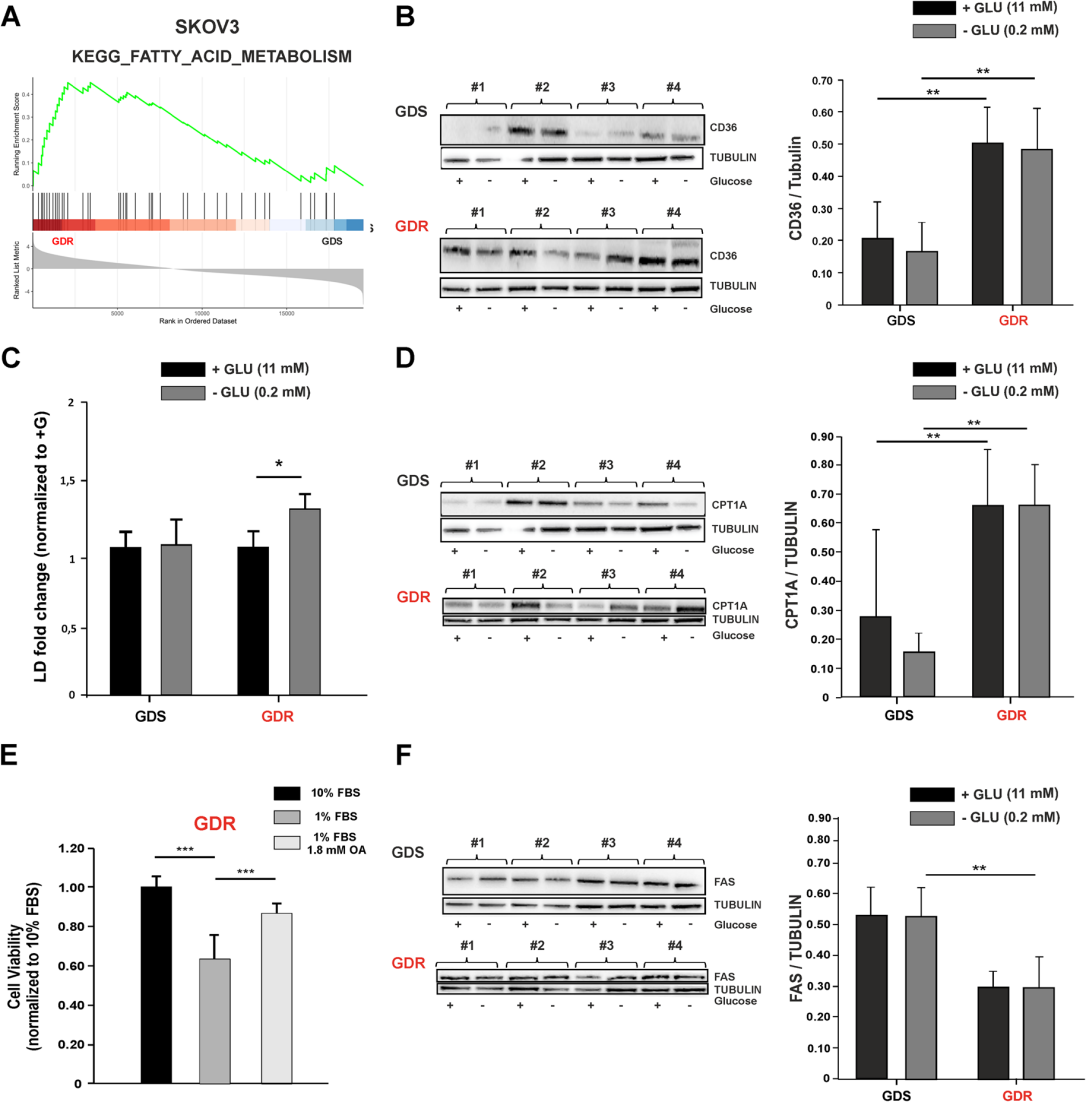
Lipid uptake and catabolism in SKOV3 GDR and GDS clones. **A** GSEA enrichment plot showing the significant upregulation of KEGG FATTY ACID METABOLISM in GDR respect to GDS clones at 24 h of glucose deprivation in the SKOV3 model. On the y-axis is showed the running enrichment score (ES) and on the x-axis the genes (vertical black lines) in the pre-ranked list belonging to the gene set. The colored band at the bottom denotes the degree of correlation of genes with the GDR phenotype (red) or the GDS phenotype (blue). **B** Western blot representing CD36 protein expression levels in SKOV3 GDS and GDR clones with or without glucose deprivation and relative quantification of CD36 abundance. Tubulin was used as loading control (4 GDS vs. 4 GDR representative clones). (**p < 0.01). **C** Lipid droplets (LD) amount in SKOV3 GDS and GDR clones measured with BODIPY staining, with or without glucose deprivation. Normalization was done on standard cell culture conditions (+ glu) (5 GDS vs. 5 GDR representative clones) (*p < 0.05). **D** Western blot representing CPT1A protein expression levels in SKOV3 GDS and GDR clones with or without glucose deprivation and relative quantification of CPT1A abundance. Tubulin was used as control (4 GDS vs. 4 GDR representative clones). (**p < 0.01). **E** Cell viability of GDR clones cultured in standard medium (10% FBS), serum-deprived medium (1% FBS) and serum-deprived medium supplemented with oleic acid (OA, 1.8 mM). Normalization was done on enriched medium culture condition (***p < 0.001). **F** Western blot representing FAS protein expression levels in SKOV3 GDS and GDR clones with or without glucose deprivation and relative quantification of FAS abundance. Tubulin was used as loading control (4 GDS vs. 4 GDR representative clones) (**p < 0.01)

Glucose deprivation triggered the significant upregulation of common processes implicated in OXPHOS both in IGROV-1 and SKOV3 GDR clones but, interestingly, fatty acid metabolism was found to be a specifically upregulated process in SKOV3 GDR clones under glucose deprivation (Fig. 5A). To functionally validate this transcriptional modulation in detail, we examined whether survival of the SKOV3 GDR clones could rely on the different regulation of FA import from the extracellular medium. CD36 protein expression (a membrane receptor involved in import of lipids) was heterogeneously expressed among GDR/GDS clones but it was not modulated by glucose limitation (Fig. 5B). Interestingly, increased expression of CD36 in GDR compared with GDS clones was observed (Fig. 5B), suggesting that GDR clones might accumulate external FAs independently from glucose concentrations in the medium. CD36 expression is positively regulated by AMPK [33] and phosphorylated (Ser79) pACC is an established AMPK target [34]. Glucose deprivation activated the AMPK pathway in SKOV3 clones, as expected, but increased CD36 expression was not associated to higher basal AMPK activation in GDR clones (Suppl. Figure 10). In view of the well-known contribution of CD36 to lipid import, we speculated that GDR cells could import more FAs from the exogenous environment, compared to GDS clones. Although no differences were found in the number of BODIPY + cells between GDS and GDR clones in presence of glucose (Fig. 5C), glucose deprivation induced a significant increase in BODIPY + cells in GDR clones respect to GDS clones, indicating an increment in lipid droplet (LD) formation (Fig. 5C). FA β-oxidation could be an important source of ATP in the absence of glucose and, in particular, we evaluated CPT1A as a key enzyme implicated in controlling fatty acid oxidation (FAO) [18]. Interestingly, a strong increase in CPT1A protein expression in GDR respect to GDS clones both in presence and absence of glucose was observed (Fig. 5D), suggesting sustained FAs β-oxidation. To investigate the potential ability of GDR clones to better exploit extracellular lipids such as serum lipoproteins and FAs contained in FBS [35], GDR clones were cultured in glucose-rich medium or glucose-poor medium supplemented with either 10% or 1% FBS. Interestingly, the reduction of FBS from 10 to 1% lowered viability (40% less) of GDR clones, as expected, but supplementation of medium with oleic acid (1.8 mM) was able to rescue this decrement (Fig. 5E). Finally, the inclination of clones to exploit *de novo* FA biosynthesis to fuel their metabolism upon glucose limitation was assessed. FAS protein expression was reduced in GDR compared to GDS clones, particularly upon glucose deprivation (Fig. 5F).

Altogether, these results suggest that GDS and GDR SKOV3 clones preferred different processes related to FA metabolism when glucose was limited in the extracellular environment, with GDR clones more prone to accumulate and metabolize external lipids, whereas GDS clones relied on accumulation and synthesis of new FAs.

### IGROV-1 GDR clones upregulate monocarboxylate transporter 1 (MCT1) and increase pyruvate import from the extracellular microenvironment upon glucose deprivation

**Fig. 6:**
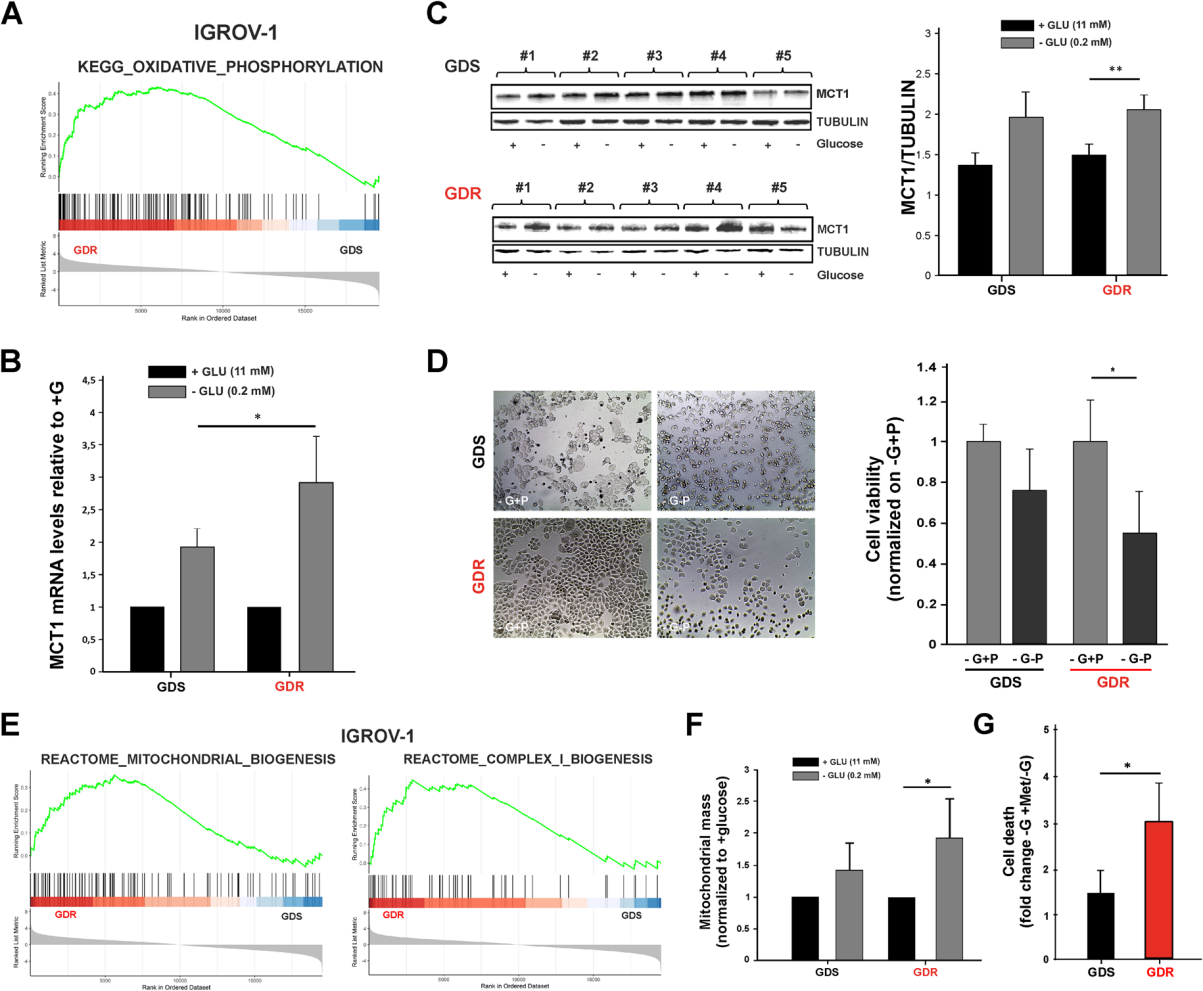
Evaluation of OXPHOS dependency in IGROV-1 GDS and GDR clones. **A** GSEA enrichment plots showing the significant upregulation of KEGG OXIDATIVE PHOSPHORYLATION PATHWAY at 24 h of glucose deprivation in IGROV-1 GDR versus GDS clones. On the y-axis the running enrichment score (ES) is showed and on the x-axis the genes (vertical black lines) in the pre-ranked list belonging to the gene set. The colored band at the bottom denotes the degree of correlation of genes with the GDR phenotype (red) or the GDS phenotype (blue). **B** MCT1 mRNA levels in IGROV-1 GDR and GDS clones under normal and glucose deprivation conditions (4 GDR and 5 GDS clones). Columns show mean values ± SD of two replicates. β2 micro globulin was used as housekeeping. (* *p* < 0.05). **C** Western blot representing MCT1 protein expression levels in SKOV3 GDS and GDR clones with or without glucose deprivation and relative quantification of MCT1 abundance. Tubulin was used as control (5 GDS vs. 5 GDR representative clones) (**p < 0.01). **D** Representative images of IGROV-1 GDS and GDR clones cultured under pyruvate and glucose starvation (left panel) and related cell viability (right panel). The graph represents the mean values ± SD of 3 GDS and 6 GDR clones after 72 h of glucose starvation (-G + P) or glucose/pyruvate starvation (-G -P) (*p < 0.05). **(E)** Gene set enrichment analysis was performed on the Reactome canonical pathways present in the Molecular Signatures Database. The enrichment plots show the significant upregulation of two Reactome pathways related to mitochondrial and complex I biogenesis in GDR vs. GDS at 24 h of glucose deprivation in the IGROV-1 model. **(F)** Mitochondrial mass of IGROV-1 GDS and GDR clones with or without glucose deprivation. Graph represents mitochondrial mass normalized on normal conditions (+ glucose). (4 GDS vs. 4 GDR clones) (*p < 0.05). **(G)** Effect of Metformin treatment on IGROV-1 GDS and GDR clones cell death under glucose starvation (5 GDR vs. 4 GDS clones). Results were represented as fold change by normalizing the values obtained with metformin treatment (1mM) for 48 h on glucose starvation condition (-G + Met/-G). (*p < 0.05)

To deepen the OXPHOS dependency of GDR clones upon glucose deprivation (Fig. 6A), we investigated the role of MCT1 in GDS and GDR clones. MCT1 controls the reversible exchange of pyruvate and lactate between the cytosol and extracellular space in cancer [36], and it is involved in pyruvate import in the mitochondria by resulting in increased OXPHOS activity [37]. A significant upregulation of MCT1 transcript expression both in SKOV3 and IGROV-1 clones under glucose starvation was observed (Suppl. Figure 11), but the highest amount of MCT1 transcript was measured in IGROV-1 GDR clones under glucose deprivation (Fig. 6B). Although upregulation of MCT1 protein expression was induced both in GDR and GDS clones upon glucose starvation, a significant increase of MCT1 protein was observed in glucose deprived GDR clones respect to GDS ones (Fig. 6C). Pyruvate can putatively be used by GDR clones to maintain cell viability under glucose starvation. Although pyruvate deprivation affected viability of IGROV-1 GDS clones, GDR clones suffered more in terms of viability when pyruvate was removed from the culture medium (Fig. 6D). In contrast, pyruvate depletion had marginal effects on SKOV3 GDR clones (Suppl. Figure 12). A further functional analysis using GSEA on Reactome canonical pathways, was useful to finely inspect the OXPHOS dependency exhibited by GDR clones upon glucose deprivation. Upregulation of mitochondrial biogenesis and complex I biogenesis pathways in IGROV-1 model was demonstrated (Fig. 6E) and, to better characterize the underlying mechanism, mitochondrial mass was used as a marker of mitochondria biogenesis [38]. Glucose deprivation was associated with an increment in mitochondrial mass both in GDS and GDR clones, but glucose-deprived GDR clones were endowed with highest mass (Fig. 6F), suggesting increased biogenesis of mitochondria as a response to glucose deprivation [39].

Finally, to target the OXPHOS metabolic dependency of GDR clones upon glucose deprivation, we employed a respiratory chain complex I inhibitor, metformin, which is known to decrease mitochondria respiration and reduce glucose metabolism through the citric acid cycle [40]. Strikingly, upon glucose starvation GDR clones were more susceptible to metformin-induced cell death when compared to GDS clones (Fig. 6G). These results confirmed the OXPHOS dependency of IGROV-1 GDR clones when glucose is limited in the tumor environment and indicate a potential therapeutic strategy to target GDR clones.

## Discussion

Low glucose and high lactate levels are common in the microenvironment of solid tumors [12] and can be exacerbated by anti-angiogenic therapy [6]. Previous studies have shown that among cancer cell lines marked differences can be found in their capability to survive glucose limitation, and recognized OXPHOS as the major pathway required for optimal proliferation under low glucose conditions [16]. In this study, we performed a comprehensive analysis of adaptation mechanisms used by clones derived from ovarian cancer cell lines to glucose limitation. Established cancer cell lines were preferred due to the feasibility to obtain cell clones, at variance with patient-derived cancer cells, whose survival and growth capacity in vitro are still limited. We identified two clonal populations, GDR and GDS, with different patterns of response to glucose limitation in vitro. We hypothesized that the GDR population could be enriched after anti-angiogenic treatment, which was known to exacerbate glucose starvation in tumors. As expected, this behaviour was observed in tumor xenografts formed by IGROV-1 and SKOV3 cells treated with bevacizumab, and also confirmed in a PDX ovarian cancer model, indicating that anti-angiogenic treatment favours survival and expansion of a glucose limitation-resistant phenotype in the tumor microenvironment.

GDR clones disclosed OXPHOS-dependent metabolic traits, compared with GDS clones and exhibited increased spare respiratory capacities, indicating higher mitochondrial reserve. These features resulted in a more adaptive metabolic phenotype enabling them to overcome glucose deprivation–derived stress and cell-death stimuli, which were instead detrimental for GDS clones [41, 42]. Several studies demonstrated that the switch from glycolysis to OXPHOS is sufficient to induce acquired resistance to drugs [43–45]. We speculate that GDR clones could be more resistant to chemotherapy, although this was not directly investigated in our study.

The higher metabolic plasticity of GDR clones compared with GDS clones emerged also from Seahorse analysis. In line with this, we observed that OXPHOS perturbations induced by oligomycin treatment triggered an increment in the extracellular amount of lactate in GDR clones, by suggesting an efficient adaptive metabolic apparatus in resisting to nutrient availability. Conversely, GDS cell metabolism appears to be less adaptive and OXPHOS blockade has relatively less impact on lactate production. The observation that OXPHOS inhibition was able to trigger a decrease in pH values in GDR clones highlighted their metabolic plasticity respect to GDS clones, by suggesting their ability to increase extracellular acidification rate in response to oligomycin treatment. This metabolic adaptation is commonly observed also in other contexts and offers a therapeutic opportunity for specifically targeting resistant cells that survive glucose deprivation. In line with previous studies [46–48], we demonstrated that GDR clones exhibited stronger sensitivity to metformin-induced cell death upon glucose deprivation when compared to GDS clones (Fig. 6G), by confirming the metabolic OXPHOS dependency observed in GDR clones when glucose is limited in the environment. These findings suggest that a combinatorial therapy by using metformin and bevacizumab could delay outgrowth of GDR clones and tumor growth. In this context, sporadic clinical observations suggest that these two drugs could indeed cooperate to cause tumor cell death [49]. The phenotypic features of GDR clones seemed not to be caused by underlying genetic alterations because we did not find relevant mitochondrial DNA mutations in these clones, at variance with Sabatini et al. who reported the presence of pathogenic mutations in mitochondrial DNA in cancer cell lines sensitive to glucose limitation [16]. Although IGROV-1 GDS clones shared a missense variant in the nuclear-encoded gene *MTG1* which was involved in mitochondrial ribosome associated GTPase1, we lack sufficient elements to attribute a functional impact of this variant on the two different GDS and GDR phenotypes and their different metabolic behaviors. Further characterization of GDR and GDS populations was conducted at the transcriptional level, in IGROV-1 and SKOV3 models. Transcriptome experiments were designed to perform comparisons over time (6 h vs. 0 h, 24 h vs. 0 h) in both clones, and comparisons between GDR and GDS at fixed time point (0 h, 6 or 24 h). Longitudinal analysis revealed massive modulation of genes and pathways under glucose deprivation for 6 or 24 h in both clonal populations. Comparative transcriptome analysis (GDR vs. GDS) confirmed the activation of an OXPHOS-related transcriptional program in GDR clones as glucose levels decreased, thus reinforcing the finding obtained at the metabolic level. Moreover, our experiments showed different patterns of activation of the same OXPHOS-related pathways in IGROV-1 and SKOV3 GDR clones, with the former characterized by rapid activation (6 h) maintained over time (24 h), while the latter by delayed activation (24 h). Despite the similar behavior noticed at the transcriptional level in IGROV-1 and SKOV3-derived GDR clones, heterogeneous putative mechanisms of metabolic adaptation to glucose limitation were observed. In particular, SKOV3 GDR clones were more prone to accumulate and metabolize external lipids when glucose was deprived, as suggested by increased expression level of CD36, a protein involved in the uptake of nutrients from the extracellular fluids [50]. On the contrary, SKOV3 GDS clones demonstrated higher propension for the synthesis of new fatty acids, as suggested by increased FAS expression levels. Although these findings might reflect cellular adaptations to in vitro experimental conditions, they corroborated the recent observation that lipid uptake and storage were increased in tumor xenografts treated with bevacizumab [51]. Along this line, Hodakoski et al. recently reported that lung cancer cells exploit Rac-mediated micropinocytosis of extracellular proteins to survive states of glucose deprivation [52], and another study reported that human pancreatic carcinoma tumor samples, which are poorly vascularized and have decreased glucose levels, undergo high rates of micropinocytosis to maintain adequate intracellular amino acid levels [13].

On the other hand, IGROV-1 GDR clones hypothetically fueled OXPHOS upon glucose deprivation by upregulating MCT1 transcript and protein, thus suggesting that pyruvate import from the extracellular microenvironment could be a fundamental process for adaptation to glucose deprivation. We indirectly confirmed the importance of this uptake in GDR clones, by demonstrating that pyruvate depletion affected viability of IGROV-1 clones under glucose deprivation. All these results suggest that adaptation to glucose starvation followed both common and different routes in SKOV3 and IGROV-1 cells, ultimately converging in the increased expression of peculiar transporters to facilitate the import of essential substrates.

The response of cancer cells to glucose starvation has been extensively investigated by several previous studies, which highlighted multiple intrinsic mechanisms, in line with results of our study [53–57]. It is also important to underline that although here we focused on clones derived from established cancer cell lines, our group previously demonstrated that GDR/GDS populations exist also in OC patient-derived samples [58], thus underscoring the translational relevance of our findings.

Finally, the underlying mechanism by which angiogenic inhibitors lead to enrichment of a clonal population resistant to glucose deprivation deserves further investigations. The possibility of targeting this population by OXPHOS inhibitors as novel therapeutic strategy could be useful to improve the efficacy of anti-angiogenic therapy in ovarian cancer.

## Conclusion

In this study, we demonstrated a metabolic heterogeneity at the clonal level in ovarian cancer cell lines by isolating different clonal populations which disclosed strikingly different viability following in vitro glucose starvation. GDR clones were enriched in experimental tumors by anti-VEGF therapy suggesting their partial involvement in the onset of resistance to glucose deprivation after anti-angiogenic drug treatment. This therapy-induced clonal population was endowed with potentially druggable OXPHOS-dependent metabolic traits and could be targeted for counteracting adaptive resistance to anti-angiogenic therapy in ovarian cancer.

### Supplementary Information

**Additional file 1: Suppl. Table 1.** Short tandem repeats (STR) analysis on IGROV-1, OC316, SKOV3, A2780, OAW42 and A2774 cell lines. **Suppl. Table 2.** Number of clones obtained from IGROV-1 and SKOV3 ex vivo cell cultures from xenografts. **Suppl. Figure 1.** Evaluation of proliferation, apoptosis and necrosis in IGROV-1 and SKOV3 GDR/GDS clones-derived tumors in vivo. **Suppl. Figure 2.** Therapy-associated enrichment of a glucose deprivation-resistant population in patient-derived xenograft (PDOVCA 62) model of ovarian cancer. **Suppl. Figure 3.** Glycolysis and OXPHOS activity in SKOV3 GDR and GDS clones. **Suppl. Figure 4.** Metabolic characteristics of GDS-enriched OC316 and GDR-enriched A2774 cells. **Suppl. Figure 5.** SNVs distribution in IGROV-1 and SKOV3 GDS and GDR clones. **Suppl. Table 3.** SNVs carried by single IGROV-1 samples or exclusively shared within a specific group. **Suppl. Table 4.** SNVs carried by single SKOV3 samples or exclusively shared within a specific group. **Suppl. Figure 6.** SNVs distribution in both IGROV-1 and SKOV3 GDS clones and GDR clones. **Suppl. Table 5.** SNVs on mitochondrial DNA of IGROV-1 clones. **Suppl. Table 6.** Differentially expressed probes upon glucose deprivation in GDR and GDS clones. **Suppl. Figure 7.** KEGG pathways significantly enriched in GDS and GDR clones upon 6 or 24 h of glucose deprivation for IGROV-1 model. **Suppl. Figure 8.** KEGG pathways significantly enriched in GDS and GDR clones upon 6 or 24 h of glucose deprivation for SKOV3 model. **Suppl. Figure 9.** Gene-concept networks of the 4 most up-regulated pathways in GDR clones. **Suppl. Figure 10.** Protein expression of the AMPK target pACC in SKOV3 GDR and GDS clones. **Suppl. Figure 11.** Real-time PCR of MCT1 gene in GDS and GDR SKOV3 clones upon glucose deprivation. **Suppl. Figure 12.** Glucose and pyruvate deprivation in GDS and GDR SKOV3 clones.

**Additional file 2. Suppl. File 1.** Metabolite abundance in GDR and GDS clones.

**Additional file 3. Suppl. File 2. **Complete GSEA results on KEGG pathways in IGROV-1 and SKOV3 models, both longitudinal (glucose deprivation condition vs. basal) and comparative (GDR vs. GDS) analysis.

## Data Availability

All data generated or analyzed during this study are included in this published article and its supplementary information files. WES and mitochondrial DNA sequencing data have been deposited in the Sequence Read Archive (SRA) under accession number PRJNA953339. Microarray data, together with the description of experiments and protocols have been deposited in the ArrayExpress database under accession number E-MTAB-12,150.

## Abbreviations

VEGF: Vascular endothelial growth factor
GDR: Glucose deprivation resistant
GDS: Glucose deprivation sensitive
OXPHOS: Oxidative phosphorylation
AMPK: 5' adenosine monophosphate-activated protein
FAs: Fatty acids
PDX: Patient derived xenograft
EOC: Epithelial ovarian cancer
FFPE: Formalin-Fixed Paraffin-Embedded
H&E: Hematoxylin and eosin
IHC: Immunohistochemistry
CD31: Cluster of differentiation 31
pHH3: Phosphorylated histone H3
OCR: Oxygen consumption rate
ECAR: Extracellular acidification rate
FCCP: Carbonyl cyanide 4- (trifluoromethoxy) phenylhydrazone
WES: Whole exome sequencing
GSEA: Gene Set Enrichment Analysis
KEGG: Kyoto Encyclopedia of Genes and Genomes
MCT1: Monocarboxylate transporter 1
MCT4: Monocarboxylate transporter 4
CD36: Cluster of differentiation 36
CPT1A: Carnitine palmitoyltransferase I
FAS: Fatty acid synthase
ACC: Acetyl-CoA carboxylase
BEVA: Bevacizumab
MVD: Microvessel density
VAF: Variant allele frequency
FAO: Fatty acid oxidation
