# Supplementary material for: Anti-VEGF therapy selects for clones resistant to glucose starvation in ovarian cancer xenografts
Source: J Exp Clin Cancer Res. 2023 Aug 7;42:196. doi: 10.1186/s13046-023-02779-x (PMC10405561; doi:10.1186/s13046-023-02779-x)
Supplement: Supplementary file 1 — Additional file 1: Suppl. Table 1. Short tandem repeats (STR) analysis on IGROV-1, OC316, SKOV3, A2780, OAW42 and A2774 cell lines. Suppl. Table 2. Number of clones obtained from IGROV-1 and SKOV3 ex vivo cell cultures from xenografts. Suppl. Figure 1. Evaluation of proliferation, apoptosis and necrosis in IGROV-1 and SKOV3 GDR/GDS clones-derived tumors in vivo. Suppl. Figure 2. Therapy-associated enrichment of a glucose deprivation-resistant population in patient-derived xenograft (PDOVCA 62) model of ovarian cancer. Suppl. Figure 3. Glycolysis and OXPHOS activity in SKOV3 GDR and GDS clones. Suppl. Figure 4. Metabolic characteristics of GDS-enriched OC316 and GDR-enriched A2774 cells. Suppl. Figure 5. SNVs distribution in IGROV-1 and SKOV3 GDS and GDR clones. Suppl. Table 3. SNVs carried by single IGROV-1 samples or exclusively shared within a specific group. Suppl. Table 4. SNVs carried by single SKOV3 samples or exclusively shared within a specific group. Suppl. Figure 6. SNVs distribution in both IGROV-1 and SKOV3 GDS clones and GDR clones. Suppl. Table 5. SNVs on mitochondrial DNA of IGROV-1 clones. Suppl. Table 6. Differentially expressed probes upon glucose deprivation in GDR and GDS clones. Suppl. Figure 7. KEGG pathways significantly enriched in GDS and GDR clones upon 6 or 24 h of glucose deprivation for IGROV-1 model. Suppl. Figure 8. KEGG pathways significantly enriched in GDS and GDR clones upon 6 or 24 h of glucose deprivation for SKOV3 model. Suppl. Figure 9. Gene-concept networks of the 4 most up-regulated pathways in GDR clones. Suppl. Figure 10. Protein expression of the AMPK target pACC in SKOV3 GDR and GDS clones. Suppl. Figure 11. Real-time PCR of MCT1 gene in GDS and GDR SKOV3 clones upon glucose deprivation. Suppl. Figure 12. Glucose and pyruvate deprivation in GDS and GDR SKOV3 clones. [file 13046_2023_2779_MOESM1_ESM.docx]

ADDITIONAL FILE 1: SUPPLEMENTARY FIGURES AND TABLES

**Anti-VEGF therapy selects for clones resistant to glucose starvation in ovarian cancer xenografts**

Daniele Boso ^1^, Martina Tognon ^2^, Matteo Curtarello ^2^, Sonia Minuzzo ^3^, Ilaria Piga ^3^, Valentina Brillo ^4^, Elisabetta Lazzarini ^1^, Jessica Carlet ^5^, Ludovica Marra ^5^, Chiara Trento ^3^, Andrea Rasola ^6^, Ionica Masgras ^6,7^, Leonardo Caporali ^8^, Fabio Del Ben ^9^, Giulia Brisotto ^9^, Matteo Turetta ^9^ , Roberta Pastorelli ^10^, Laura Brunelli ^10^, Filippo Navaglia ^11^, Giovanni Esposito ^2^, Angela Grassi ^2^, Stefano Indraccolo ^1,3,*^ .

1. Basic and Translational Oncology Unit, Veneto Institute of Oncology IOV-IRCCS, Padova, Italy

2. Immunology and Molecular Oncology Unit, Veneto Institute of Oncology IOV-IRCCS, Padova, Italy

3. Department of Surgery, Oncology and Gastroenterology, University of Padova, Italy

4. Department of Biology, University of Padova, Padova, Italy.

5. Medical Oncology 2, Veneto Institute of Oncology IOV-IRCCS, Padova, Italy.

6. Department of Biomedical Sciences, University of Padova, Padova, Italy.

7. Institute of Neuroscience, National Research Council, Padova, Italy.

8. Department of Biomedical and Neuromotor Sciences - DIBINEM, University of Bologna, Bologna, Italy.

9. Immunopathology and Cancer Biomarkers, Centro di Riferimento Oncologico di Aviano (CRO)-IRCCS, Aviano, Italy.

10. Laboratory of Mass Spectrometry, Department of Environmental Health Sciences, Istituto di Ricerche Farmacologiche Mario Negri IRCCS, Milan, Italy.

11. Laboratory Medicine, Department of Medicine-DIMED, University of Padova, Padova, Italy.

**Corresponding author**

**Stefano Indraccolo**

Affiliation 1: Basic and Translational Oncology Unit, Veneto Institute of Oncology IOV-IRCCS, via Gattamelata 64, Padova, Italy 35128;

Affiliation 2: Department of Surgery, Oncology and Gastroenterology, University of Padova, via Giustiniani 2, Padova, Italy, 35124, Phone: +390498215875, e-mail: stefano.indraccolo@unipd.it

**
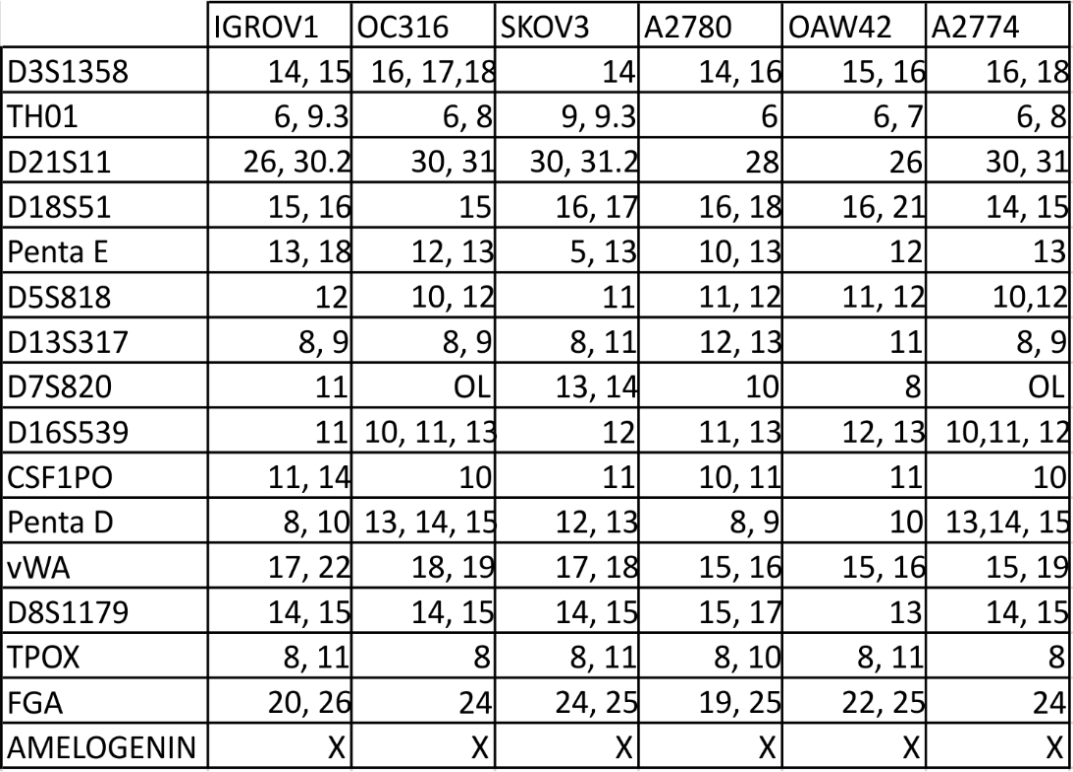
**

**Suppl. Table 1. Short tandem repeats (STR) analysis on IGROV-1, OC316, SKOV3, A2780, OAW42 and A2774 cell lines.** OL: off-ladder alleles


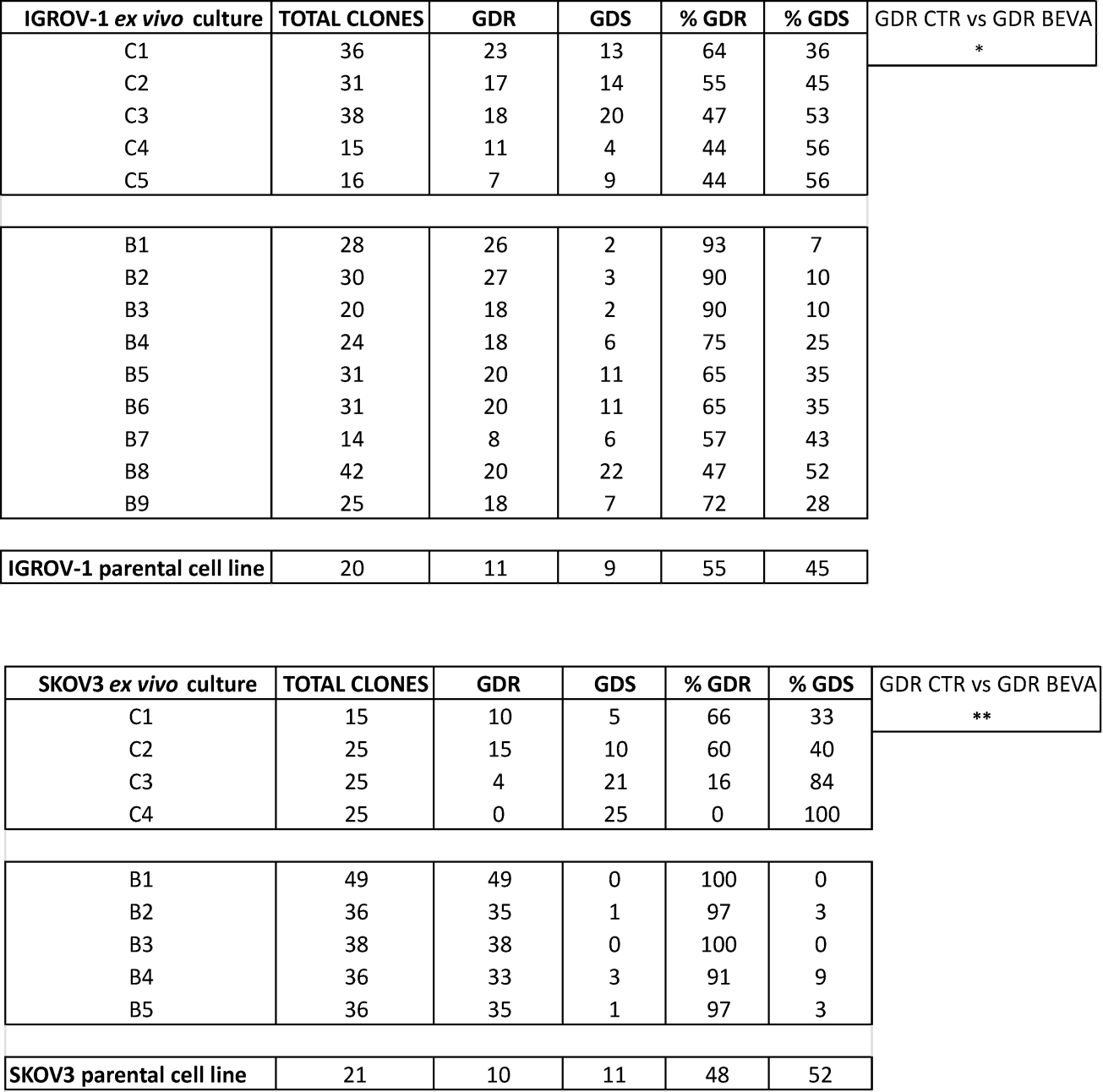


**Suppl. Table 2. Number of clones obtained from IGROV-1 and SKOV3 *ex vivo* cell cultures from xenografts.** Number of clones obtained from IGROV-1 and SKOV3 *ex vivo* cell cultures from xenografts. Percentage was calculated as number of GDR or GDS clones on number of total clones. T test was performed between GDR from CTR tumors and GDR from BEVA tumors. (*****p=0.0158;******p=0.0036)

<
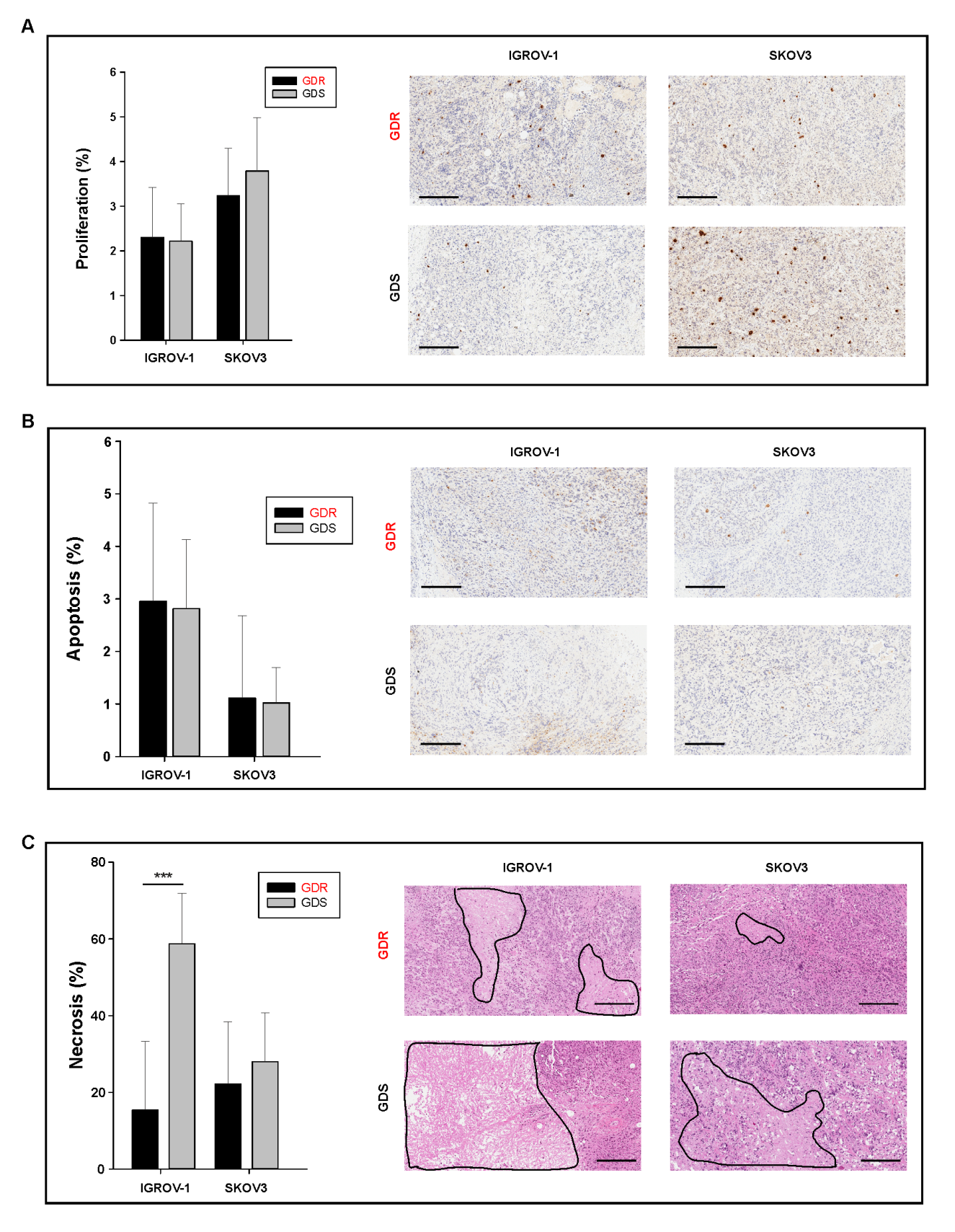


**Suppl. Figure 1. Evaluation of proliferation, apoptosis** **and necrosis in IGROV-1 and SKOV3 GDR/GDS clones-derived tumors *in vivo*. A**-**C** Evaluation of proliferation, apoptosis and necrosis processes based on immunohistochemical (IHC) expression of phospho-Histone H3 (pHH3), caspase 3 and quantification of necrotic areas (n=8 different tumors for each group), respectively (Scale bar=200µM). The slides were digitally acquired at ×10 magnification by the Aperio CS2 (Leica Biosystems, Wetzlar, Germany), and quantification (left panels) was obtained through the Scanscope Image Analysis software (ImageScope v12.4.0.708). Columns show mean± SD values (n = 4 tumors for group). Representative histological images are depicted in the right panels. Original magnification 1.25x.***p < 0.001, t-test.


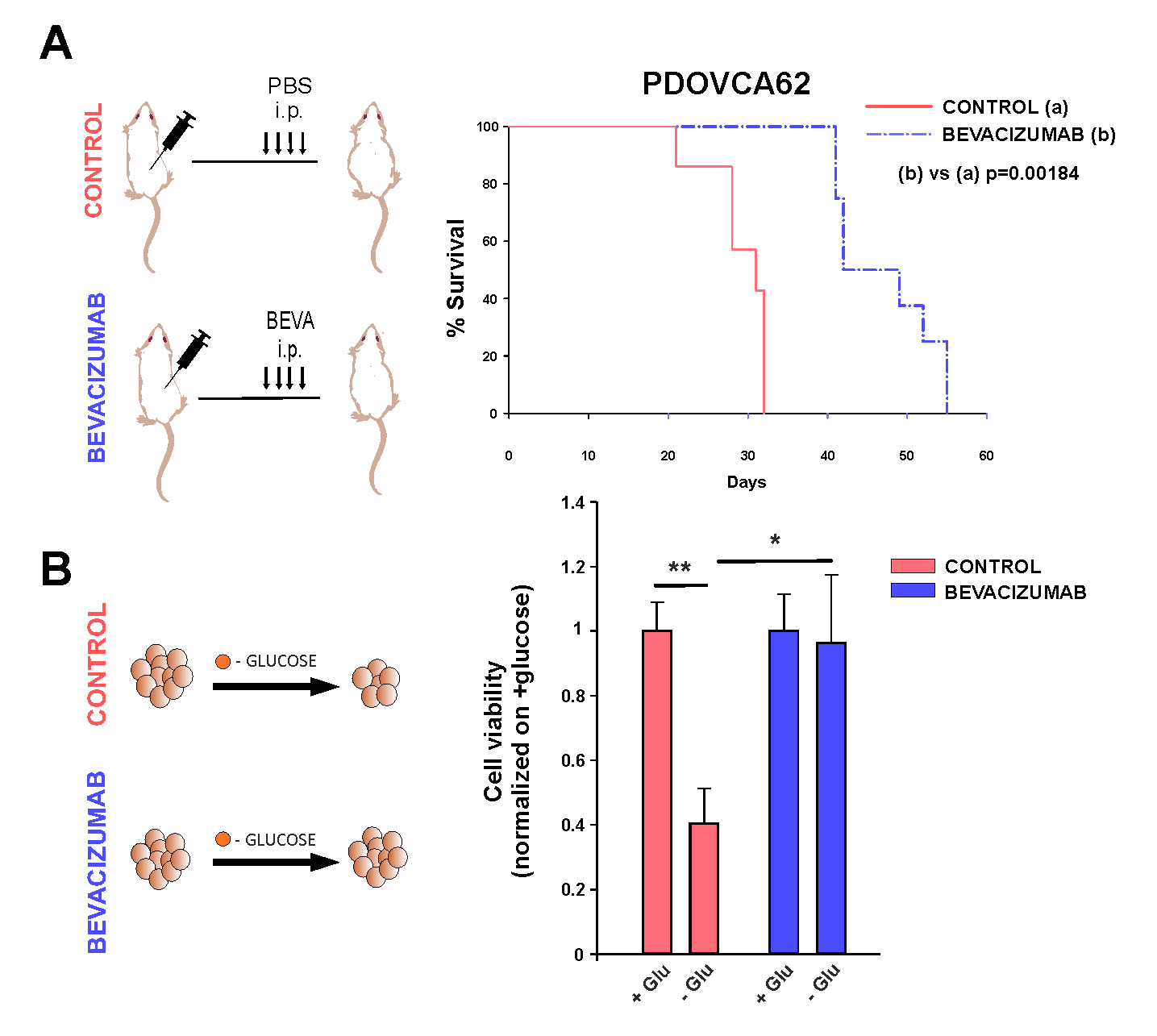


**Suppl. Figure 2. Therapy-associated enrichment of a glucose deprivation-resistant population in patient-derived xenograft (PDOVCA 62) model of ovarian cancer**. **A.** Schematic representation of ovarian cancer PDX development and bevacizumab treatment response. PDOVCA 62 PDXs were injected intra peritoneally (i.p) in NOD/SCID mice and bevacizumab treatment was applied twice per week until sacrifice (left panel). Kaplan-Meier survival curves of mice engrafted with PDOVCA 62 after bevacizumab treatment (right panel) (n=7 CONTROL mice, n=8 BEVACIZUMAB mice) (log-rank test p=0.00184). **B.** PDOVCA 62 cancer cells were isolated from PDX ascites and cultured under glucose deprivation (left panel). Cell viability measurement of PDOVCA 62 cancer cells cultured in normal condition or upon glucose deprivation (right panel). Normalization was done on normal culture condition (+glucose). (n=3 Control vs 3 Bevacizumab) (*p<0.05; **p < 0.01).


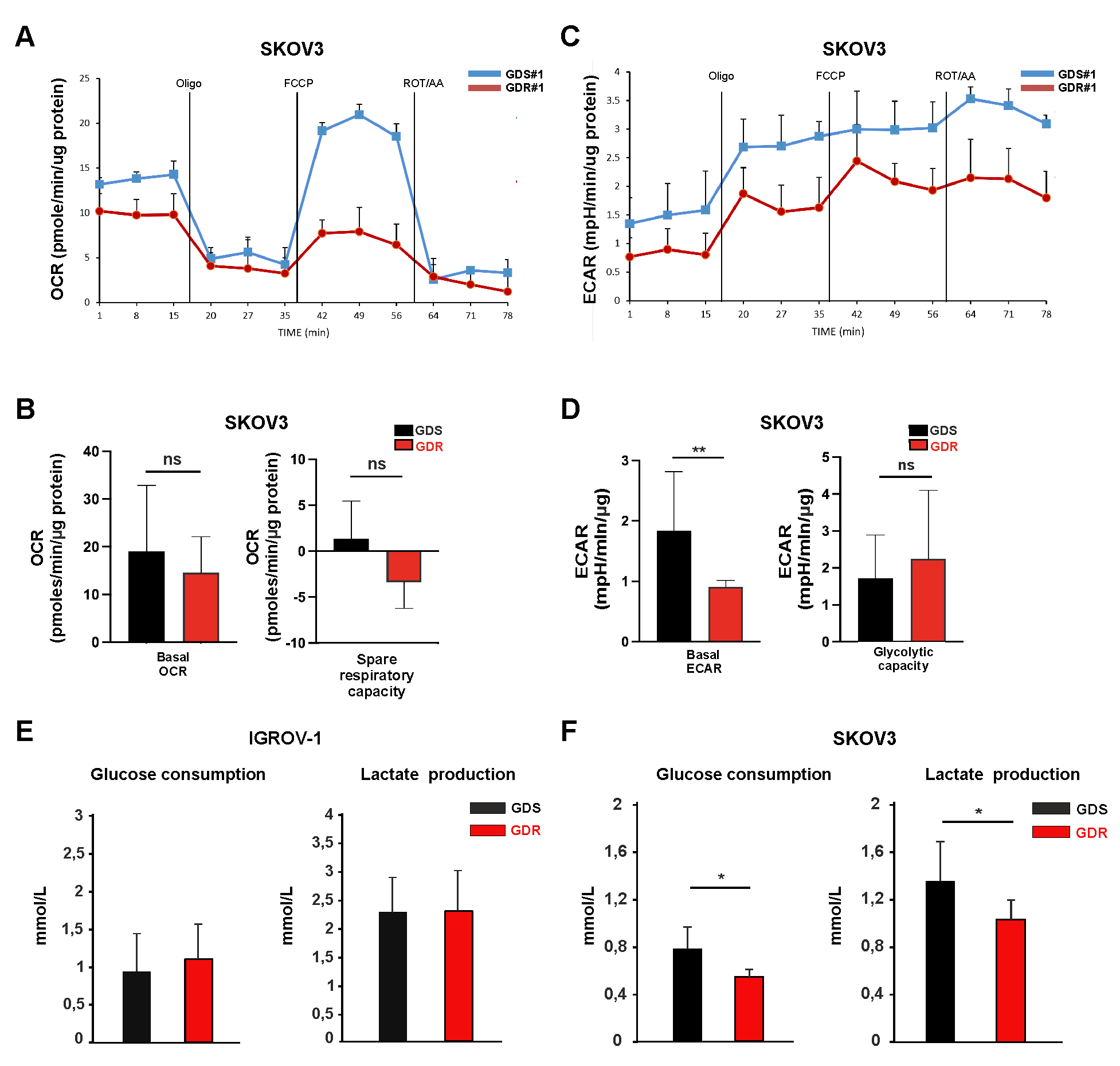


**Suppl. Figure 3. Glycolysis and OXPHOS activity in SKOV3 GDR and GDS clones.** **A**. Seahorse measurements of oxygen consumption rate (OCR) of representative SKOV3 GDR and GDS clones (GDS#1, GDR#1). Four different metabolic inhibitors were administered at 20 minutes intervals: oligomycin (1μM), followed by carbonyl cyanide trifluoromethoxyphenylhydrazone, (FCCP, 0.4 μM), antimycin (1μM) and rotenone (1μM) over 2 hours. **B.** Basal respiration (left panel) and spare respiratory capacity (right panel) of SKOV3 GDR and GDS clones (3 GDS vs 4 GDR) (ns: not significant). **C.** Seahorse measurements of extracellular acidification rate (ECAR) of the same representative SKOV3 GDR and GDS clones (GDS#1, GDR#1). **D.** Basal ECAR (left panel) and maximal glycolytic capacity (right panel) of SKOV3 GDR and GDS clones (3 GDS vs 4 GDR) (ns: not significant). **E, F.** Measurement of glucose consumption and lactate production in IGROV-1 and SKOV3 GDR and GDS clones. Data are expressed as mean values (± SD) for n=15 GDR and n=9 GDS IGROV-1 clones, whereas for GDR and GDS (n=4 vs 4, glucose consumption; n=5 vs 5, lactate production) SKOV3 clones (Mann-Whitney test *p< 0.05).


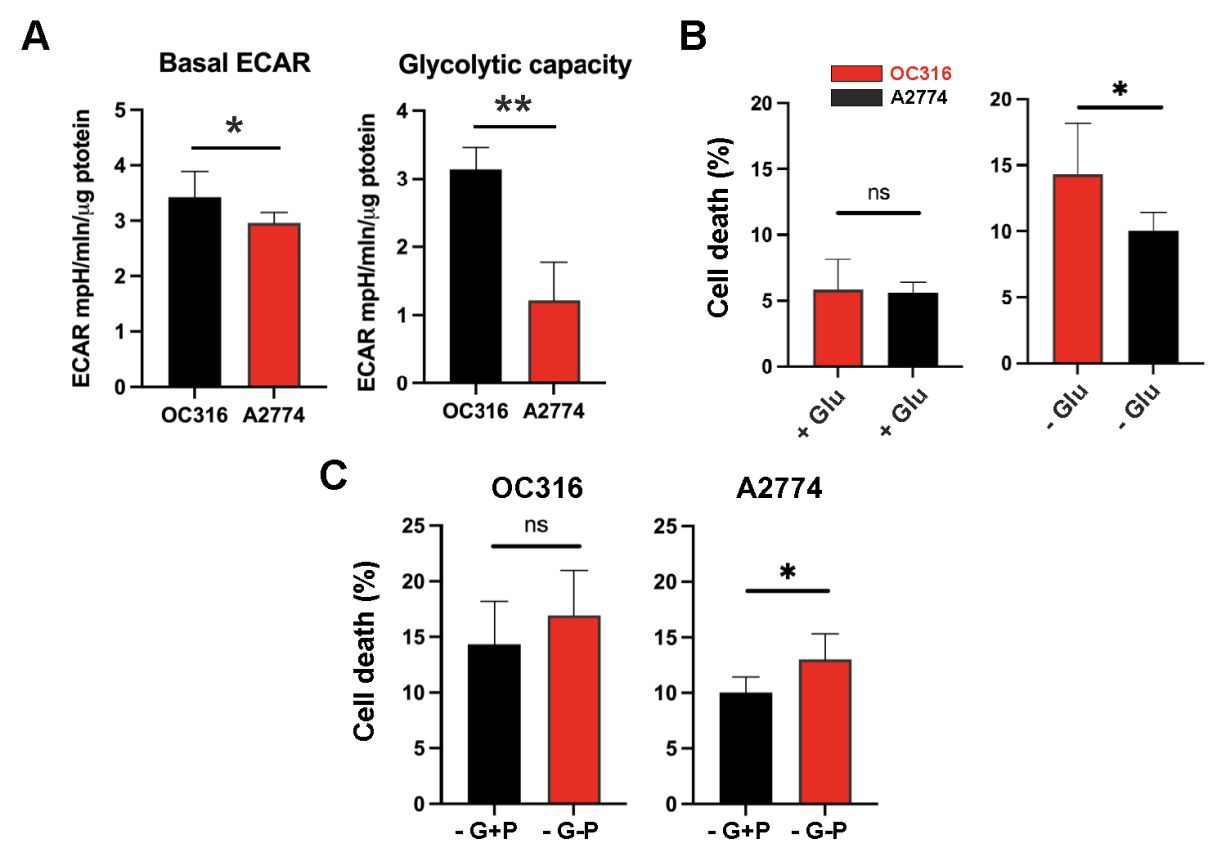


**Suppl. Figure 4. Metabolic characteristics of GDS-enriched OC316 and GDR-enriched A2774 cells. A.** Seahorse measurements of basal extracellular acidification rate (ECAR) and maximal glycolytic capacity of OC316 and A2774 cells (n=9, unpaired Student's *t* test: *p< 0.05). **B.** OC316 and A2774 were cultured either in standard (+glu) or low glucose (-glu) medium and, after 72 hours, cell death was measured by using Annexin V/PI staining (n=6, unpaired Student's *t* test, ns: not significant, *p < 0.05) **C.** OC316 (left panel) and A2774 (right panel) were cultured under glucose (-G +P) or glucose/pyruvate starvation (-G -P) and, after 72 hours, cell death was measured by using Annexin V/PI staining (n=6, unpaired Student's *t* test *p<0.05).

**
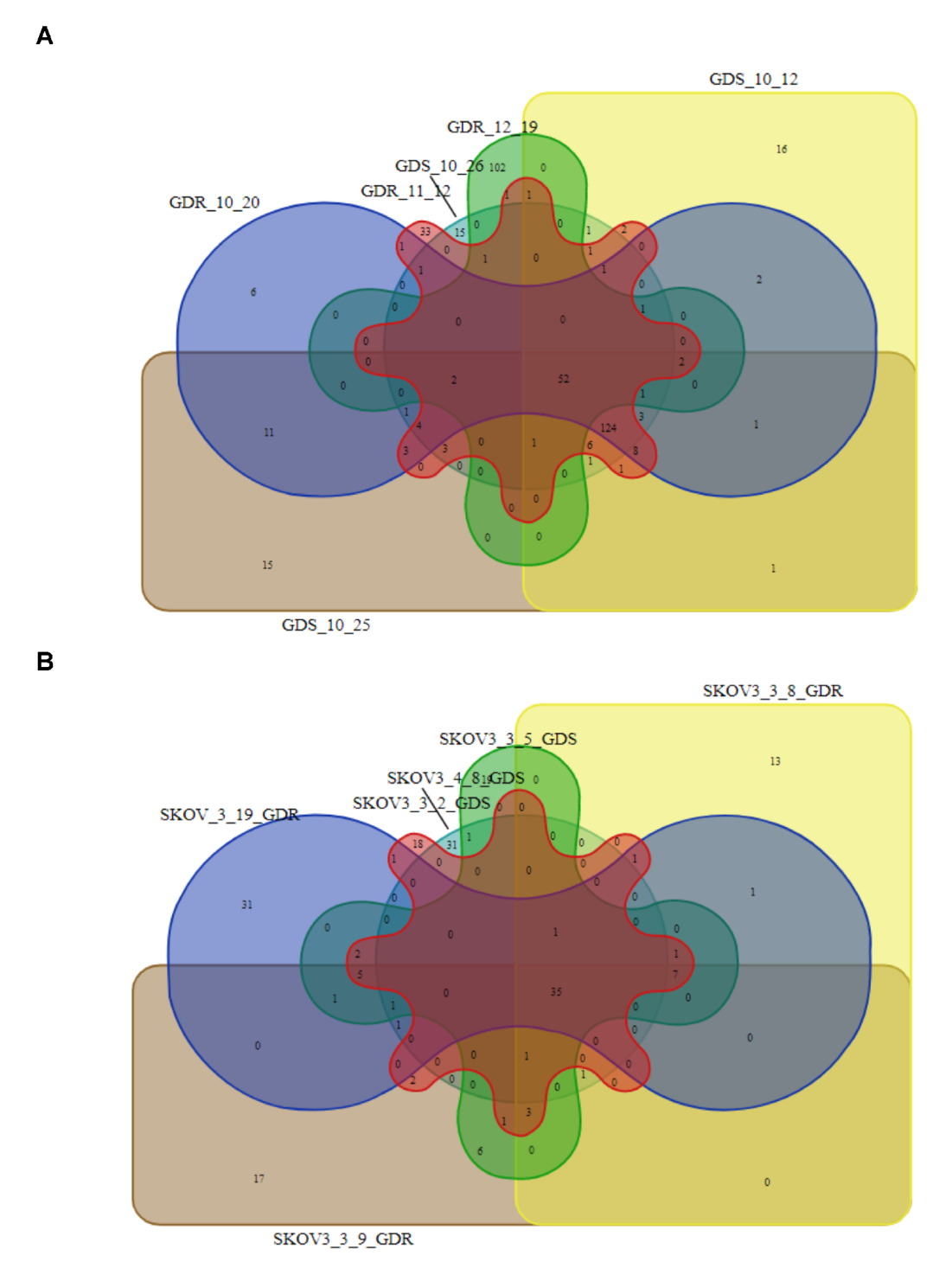
**

**Suppl. Figure 5. SNVs distribution in IGROV-1 and SKOV3 GDS and GDR clones**. **A.** Number of SNVs in 3 different IGROV-1 GDR (GDR_10_20, GDR_11_12, GDR_12_19) and 3 different GDS (GDS_10_12, GDS_10_25, GDS_10_26) clones. **B.** Number of SNVs in 3 different SKOV3 GDR (GDR_3-9, GDR_3-8, GDR_3-19) and 3 GDS (GDS_3-2, GDS_4-8, GDS_3-5) clones. Venn diagrams were created by using a free website tool (https://bioinformatics.psb.ugent.be/webtools/Venn/).

**
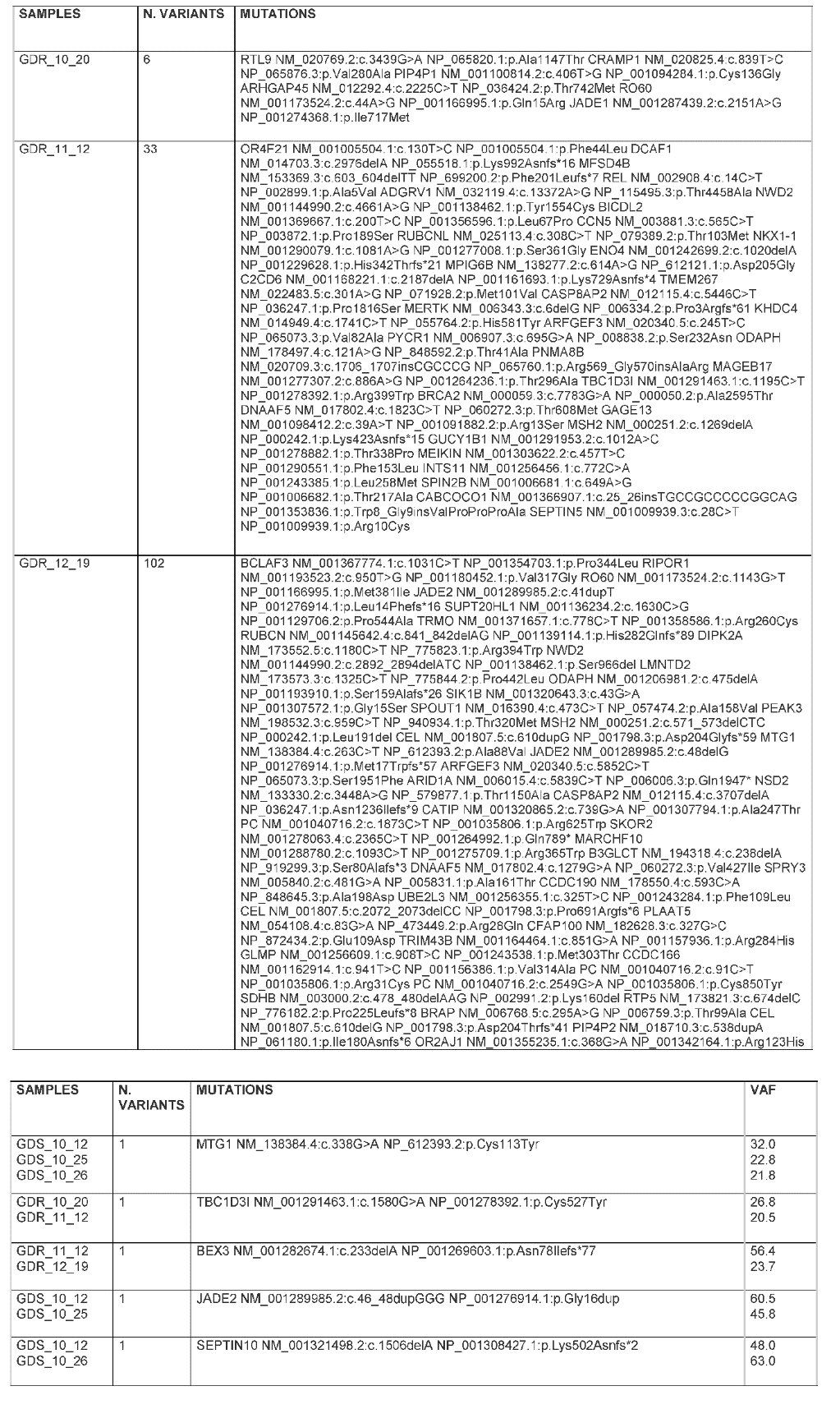
**

**Suppl. Table 3.** SNVs carried by single IGROV-1 samples or exclusively shared within a specific group (variant allele frequency of every variant is >20%).


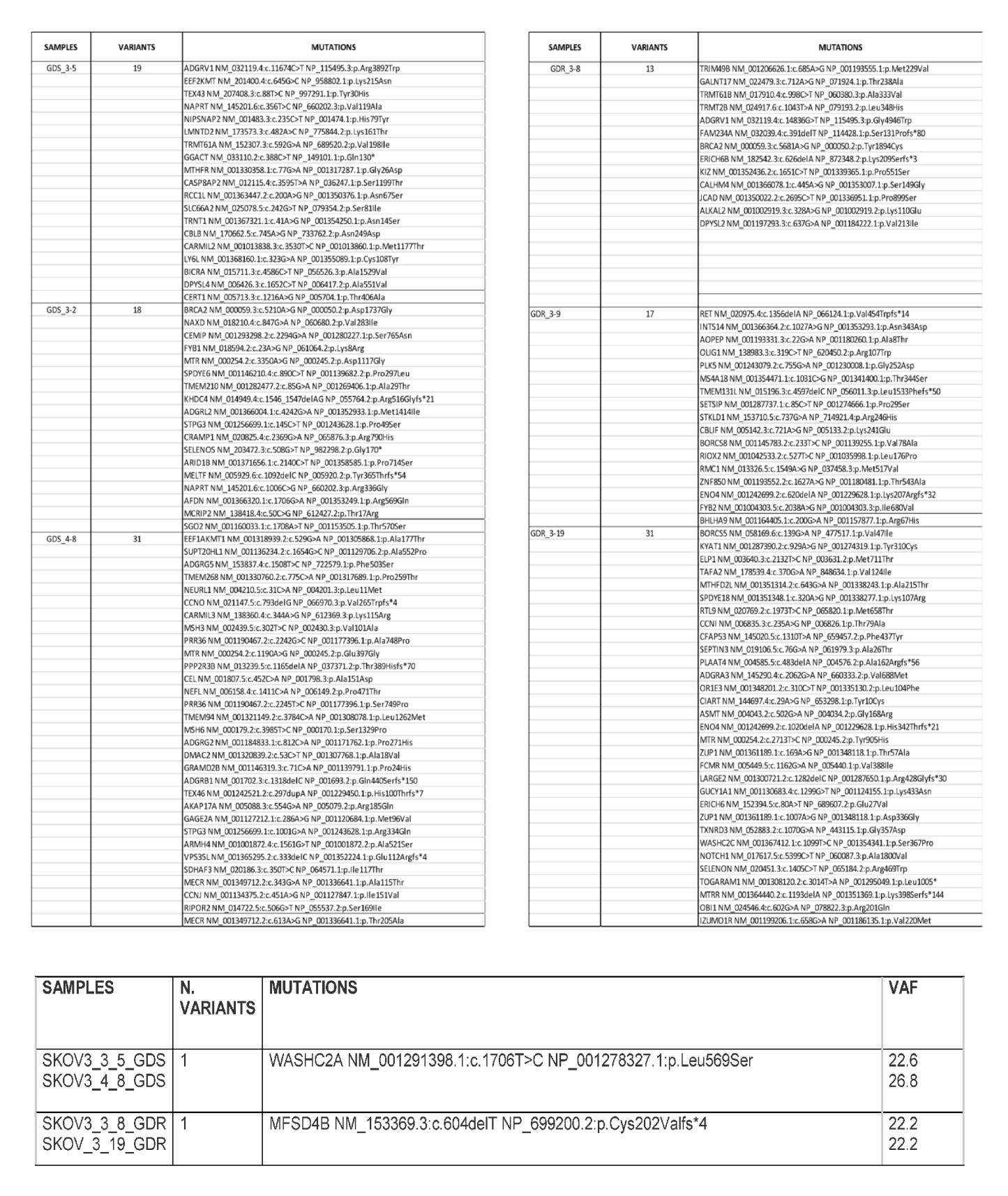


**Suppl. Table 4.** SNVs carried by single SKOV3 samples or exclusively shared within a specific group (variant allele frequency of every variant is >20%).

**
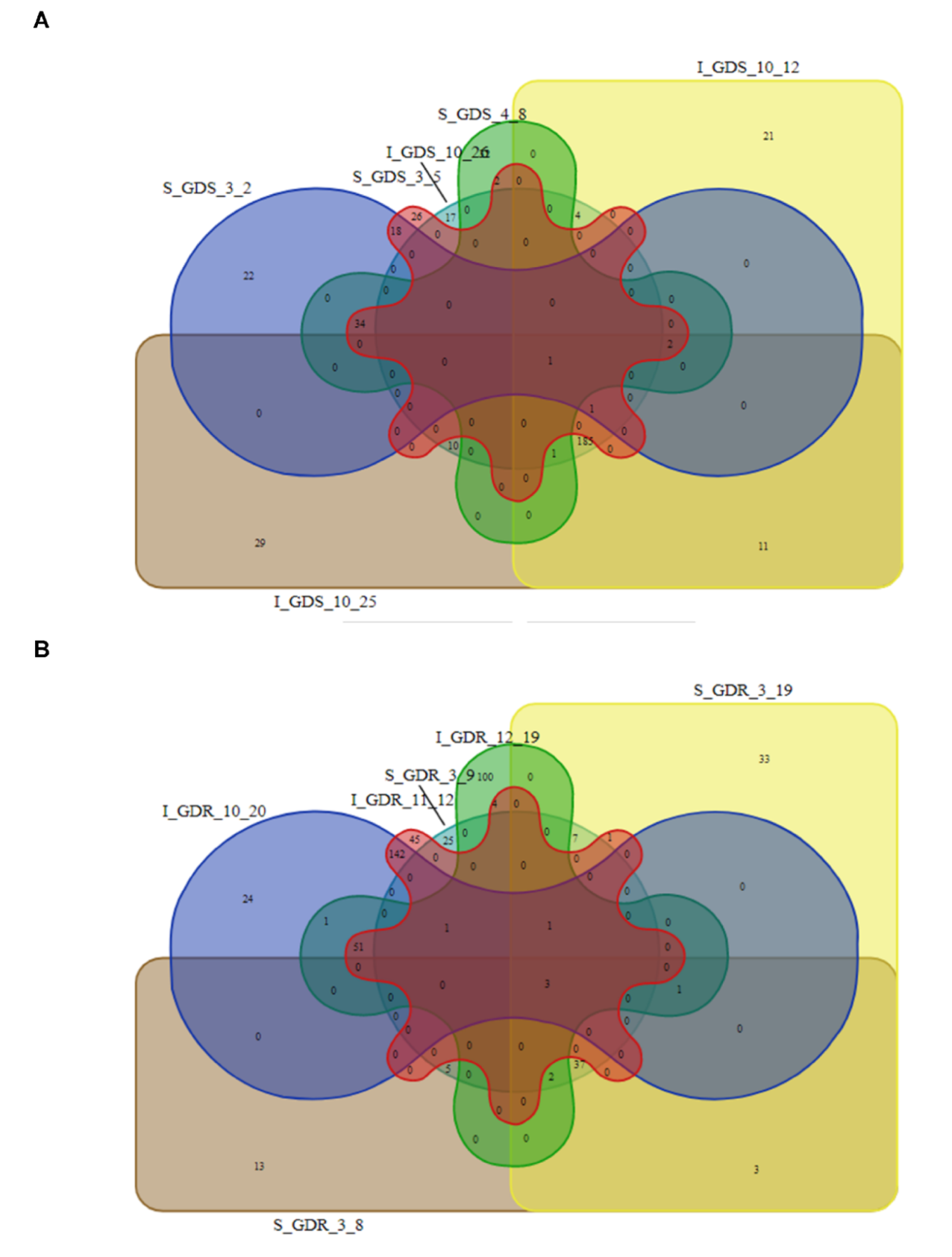
**

**Suppl. Figure 6. SNVs distribution in both IGROV-1 and SKOV3 GDS clones and GDR clones**. **A.** Number of SNVs in both IGROV-1 and SKOV3 GDS clones. **B.** Number of SNVs in both IGROV-1 and SKOV3 GDR clones.


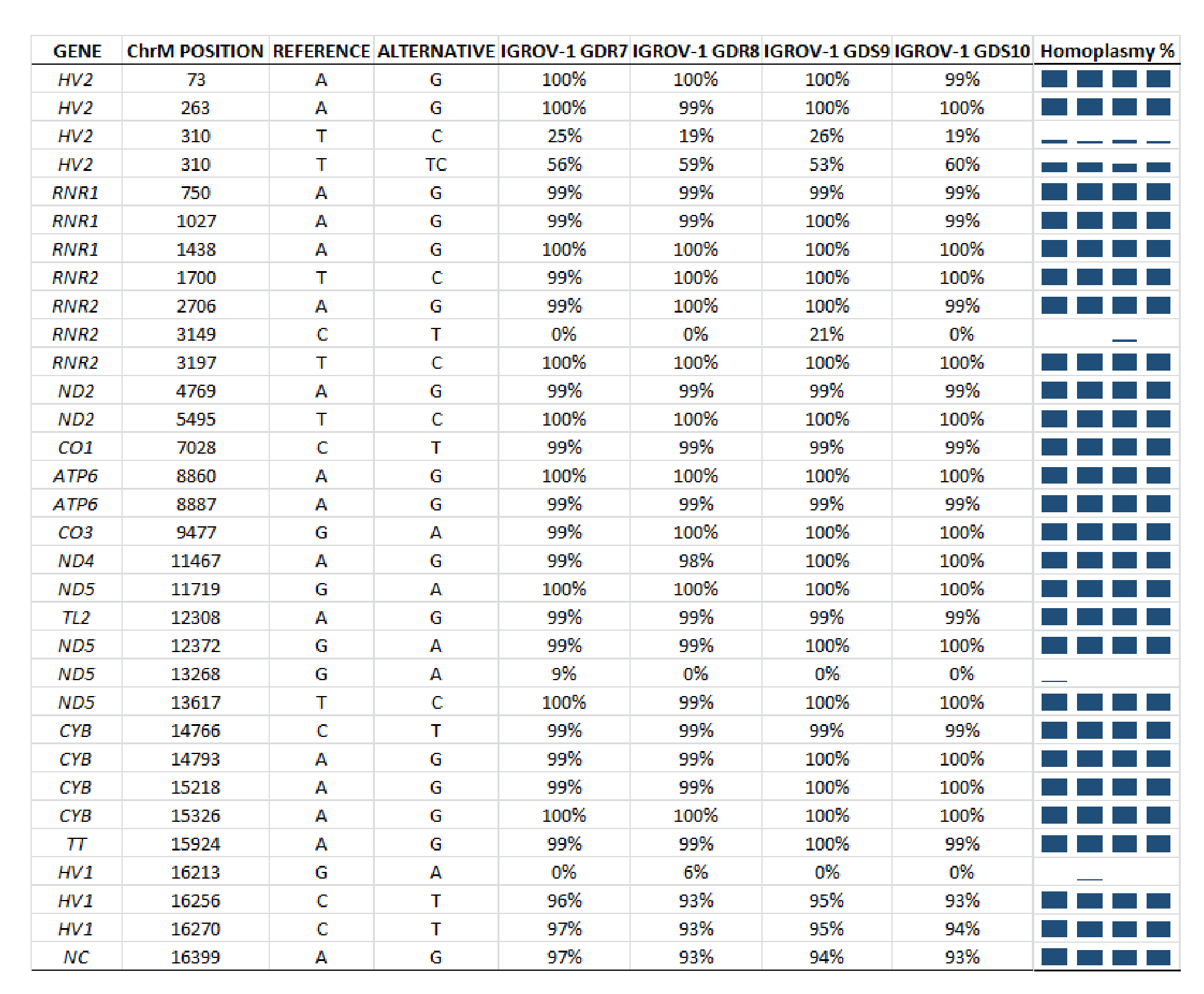


**Suppl. Table 5.** SNVs on mitochondrial DNA of IGROV-1 clones.

|  | **IGROV-1 model** | | | **SKOV3 model** | | |
| --- | --- | --- | --- | --- | --- | --- |
| **COMPARISON** | **DOWN** | **UP** | **DE probes** | **DOWN** | **UP** | **DE probes** |
| GDR 6h vs. GDR 0h | 469 | 267 | **736** | 56 | 196 | **252** |
| GDR 24h vs. GDR 0h | 6834 | 6891 | **13725** | 4465 | 4891 | **9356** |
| GDS 6h vs. GDS 0h | 1336 | 1068 | **2404** | 490 | 896 | **1386** |
| GDS 24h vs. GDS 0h | 7710 | 7642 | **15352** | 4151 | 4603 | **8754** |
| GDR 0h vs. GDS 0h | 1 | 0 | **1** | 0 | 1 | **1** |
| GDR 6h vs. GDS 6h | 0 | 0 | **0** | 0 | 0 | **0** |
| GDR 24h vs. GDS 24h | 15 | 7 | **22** | 0 | 0 | **0** |

**Suppl. Table 6. Differentially expressed probes upon glucose deprivation in GDR and GDS clones.** Differential expression analysis results in terms of number of probes significantly modulated in IGROV-1 and SKOV3 clones (both GDR and GDS) upon glucose deprivation. All the comparisons performed are indicated in the first column. Transcriptional activation of GDR or GDS clones was evaluated over time, comparing the glucose deprivation condition (6 h or 24 h) with the basal condition (0 h). A direct comparison of GDR and GDS clones at the same time point was also performed. Significant probes were determined using a cut-off of 0.01 on Benjamini-Hochberg adjusted p-value. DOWN: downregulated, UP: upregulated, DE: differentially expressed.

**
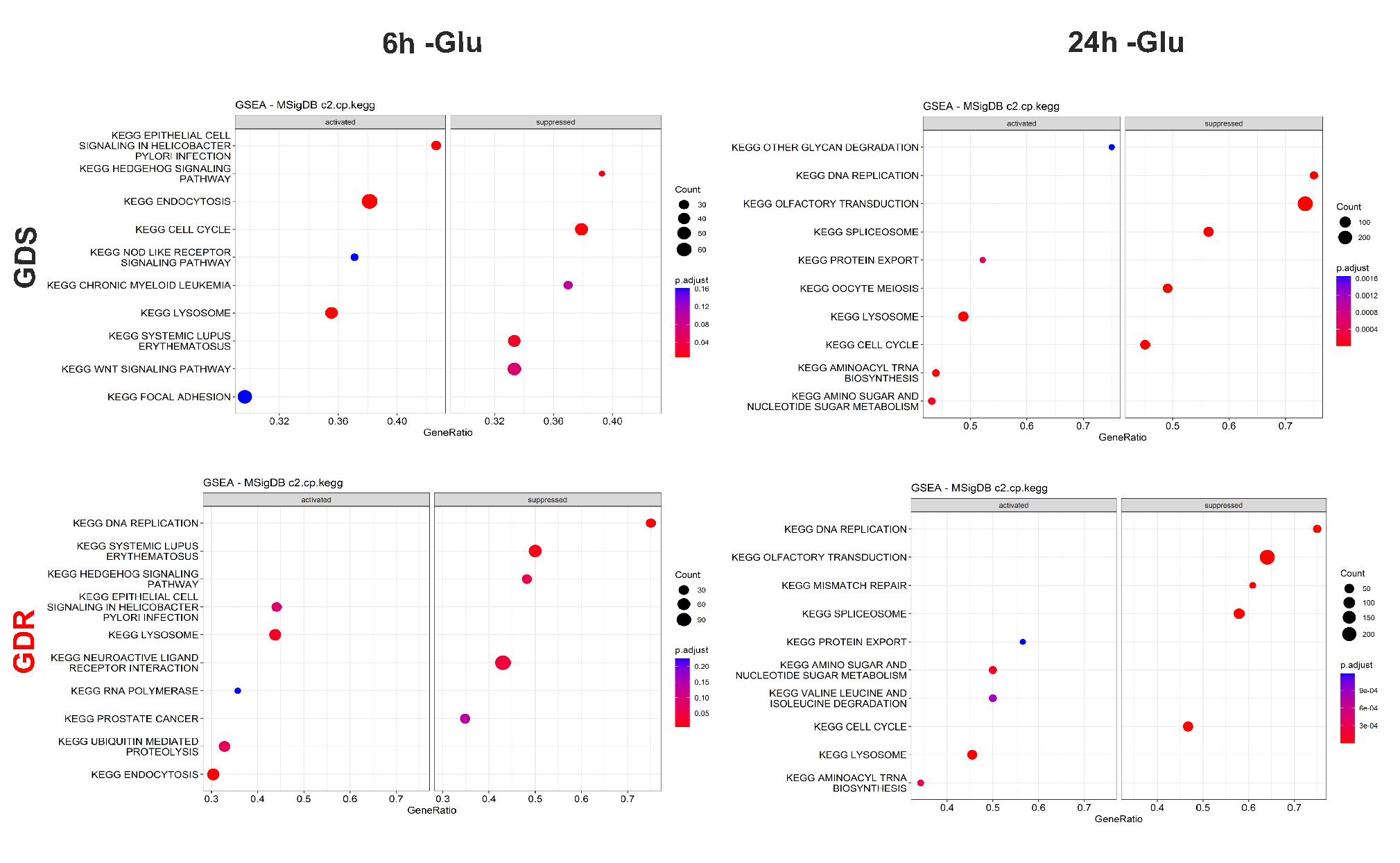
**

**Suppl. Figure 7. KEGG pathways significantly enriched in GDS and GDR clones upon 6 or 24 hours of glucose deprivation for IGROV-1 model.** Dot plots showing the results of gene set enrichment analysis (GSEA) on KEGG pathways. For each condition, the 5 most significantly activated and suppressed pathways are displayed. The size of each dot (count) represents the number of genes contributing to the enrichment of that pathway, and the color represents the Benjamini-Hochberg adjusted p-value (red dots are the most significant). Enriched KEGG pathway are ordered by decreasing gene ratio (count/size of the gene set).


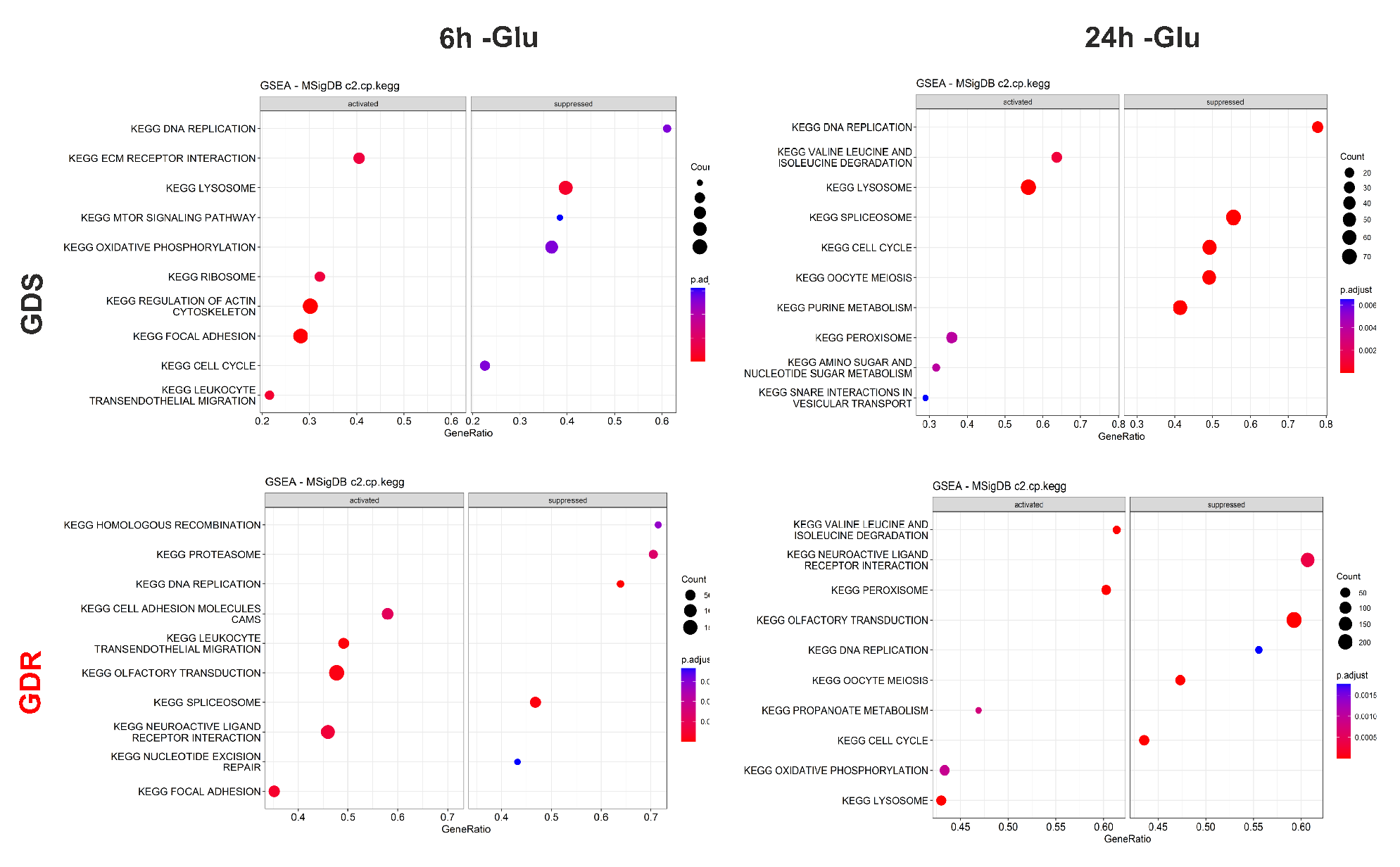


**Suppl. Figure 8. KEGG pathways significantly enriched in GDS and GDR clones upon 6 or 24 hours of glucose deprivation for SKOV3 model.** Dot plots showing the results of gene set enrichment analysis (GSEA) on KEGG pathways. For each condition, the 5 most significantly activated and suppressed pathways are displayed. The size of each dot (count) represents the number of genes contributing to the enrichment of that pathway, and the color represents the Benjamini-Hochberg adjusted p-value (red dots are the most significant). Enriched KEGG pathway are ordered by decreasing gene ratio (count/size of the gene set).

**
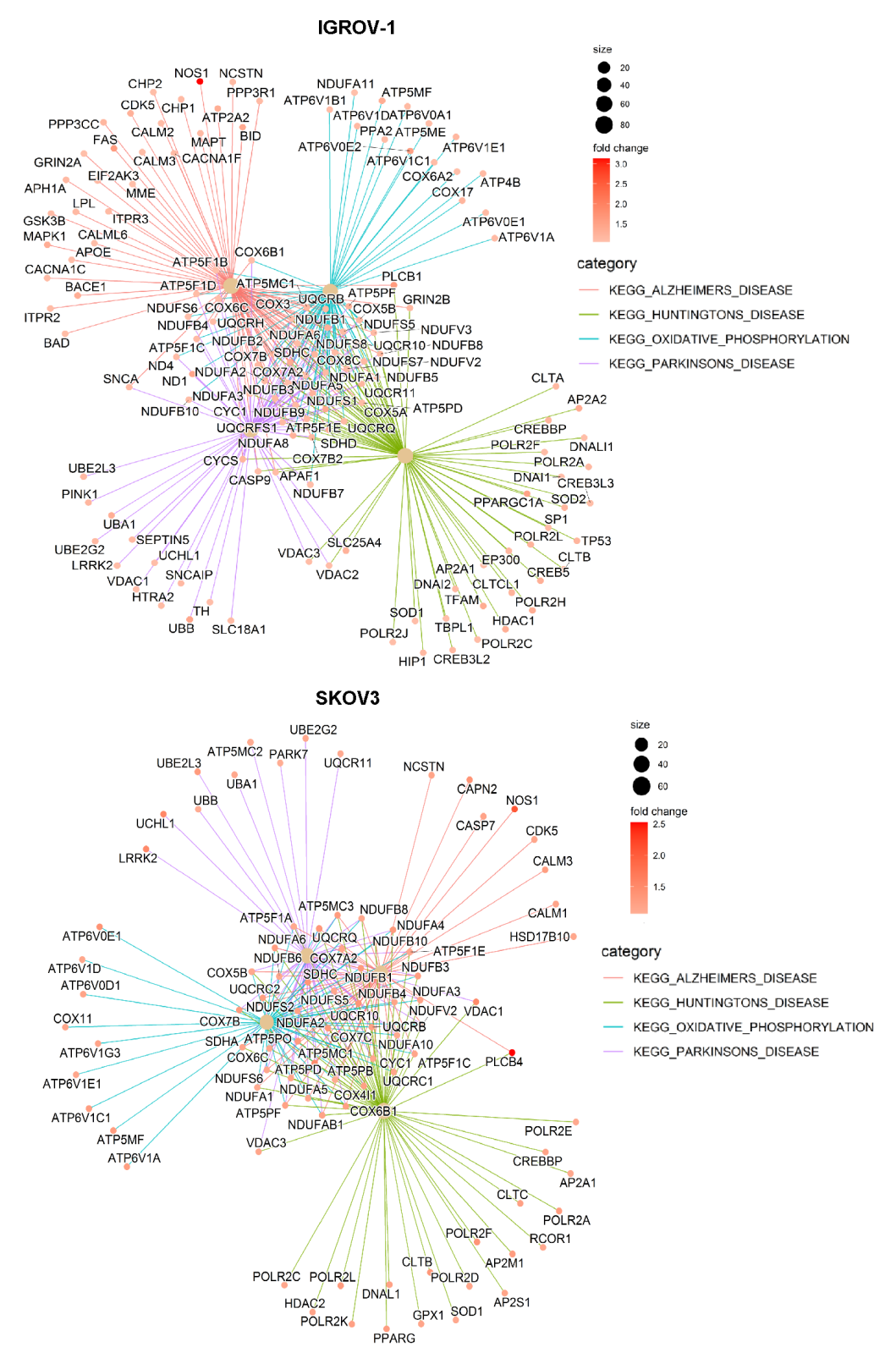
**

**Suppl. Figure 9. Gene-concept networks of the 4 most up-regulated pathways in GDR clones.** Interconnections among core genes contributing to the positive enrichment of the four most upregulated pathways in both IGROV-1 and SKOV3 GDR clones after 24 hours of glucose deprivation were visualized (adjusted p <0.0005).


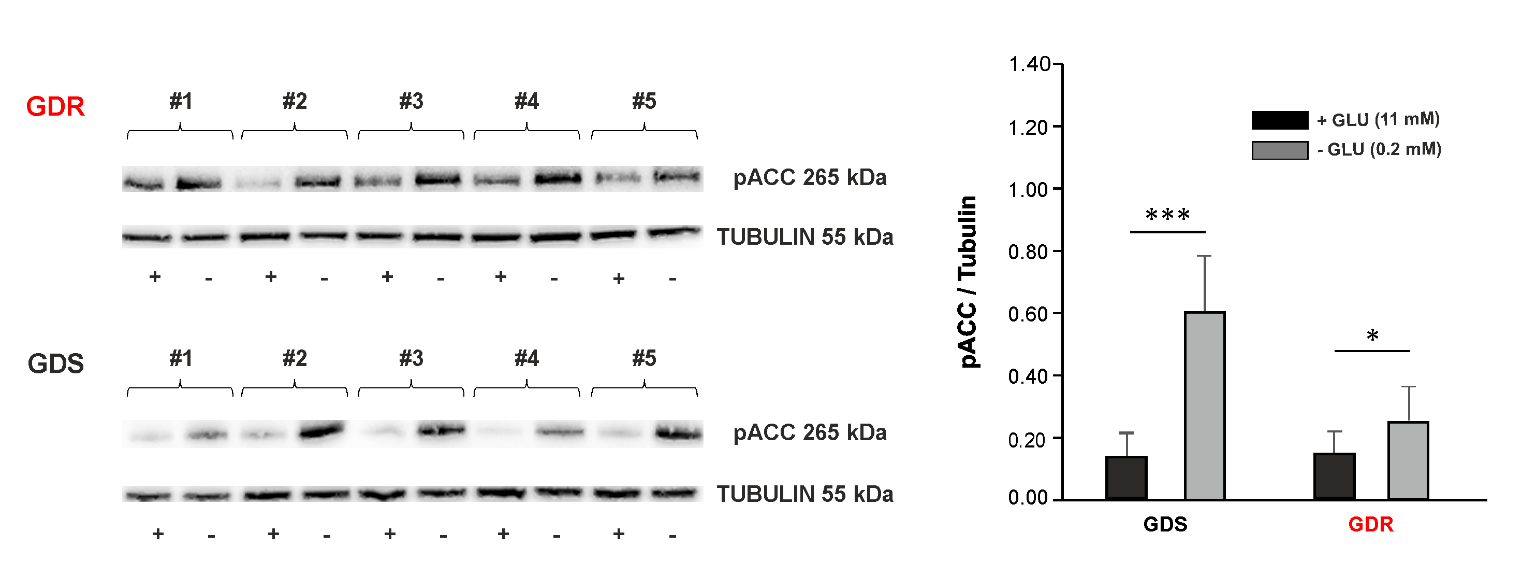


**Suppl. Figure 10. Protein expression of the AMPK target pACC in SKOV3 GDR and GDS clones.** Western blot analysis of the AMPK target pACC protein SKOV3 GDR and GDS clones. GDS and GDR clones were cultured in normal culture conditions (+) or upon glucose deprivation (-) for 48 hours. In the figure are shown mean value ± SD of 5 GDR and 5 GDS representative clones. Quantification data was obtained by normalizing pACC expression against α-tubulin. Statistical analysis was performed by using the unpaired Student's *t* test (two-tailed): *p<0.05, ***p<0.001.

**
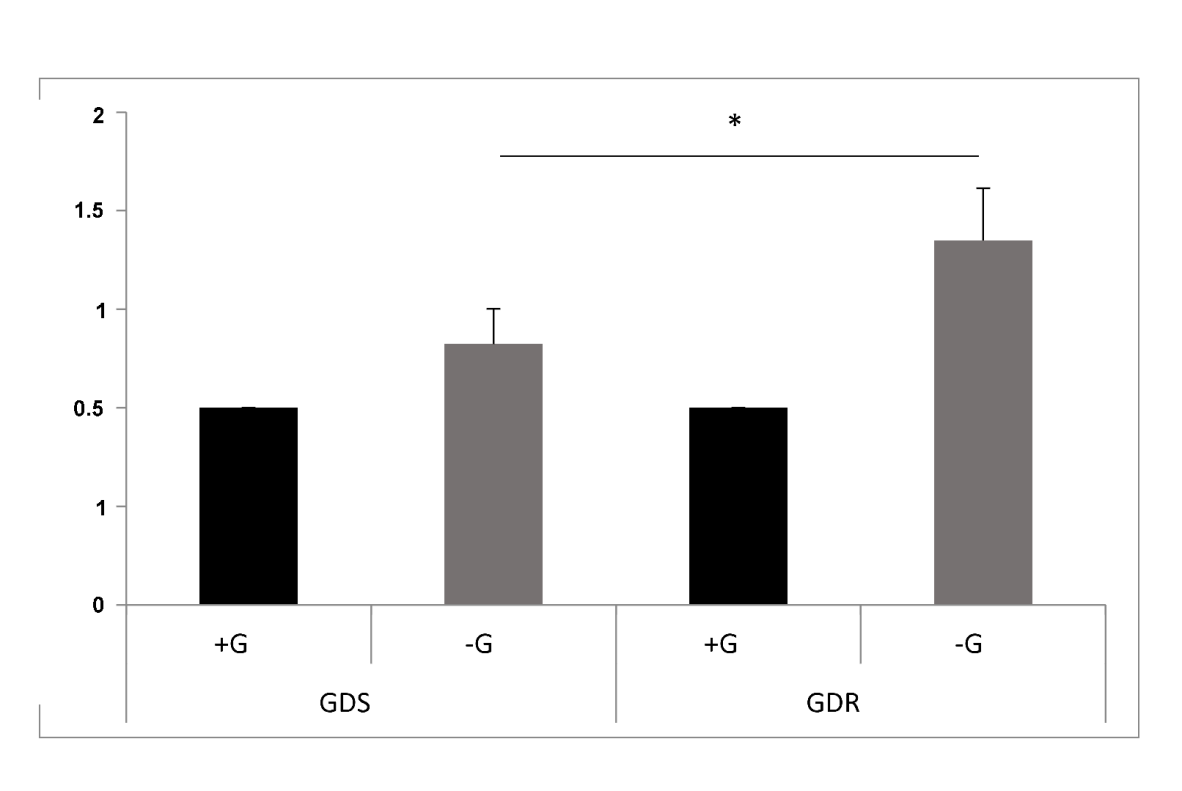
**

**Suppl. Figure 11. Real-time PCR of MCT1 gene in GDS and GDR SKOV3 clones upon glucose deprivation.** mRNA levels of MCT1 gene were measured in SKOV3 GDS and GDR clones in normal culture condition (+G) and upon glucose deprivation (-G) by using a relative quantification method. (4 GDS vs 4 GDR clones). (*p<0.05).

**
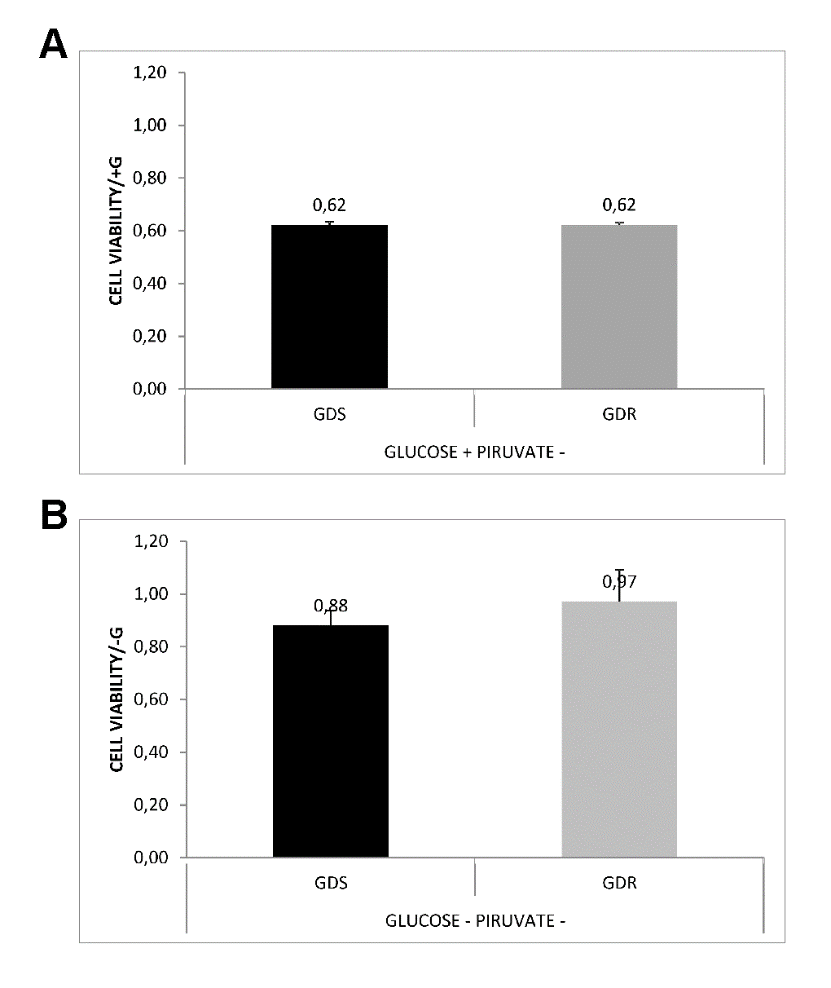
**

**Suppl. Figure 12. Glucose and pyruvate deprivation in GDS and GDR SKOV3 clones.** Cell viability of SKOV3 GDS and GDR clones cultured under pyruvate starvation **(A)** and glucose/pyruvate starvation **(B)**. The graphs represent the mean values ± SD of 5 GDR and 5 GDS clones after 72 hours of glucose starvation (-G +P) or glucose/pyruvate starvation (-G -P). Cell viability was measured by using Sulforhodamine B Assay Kit (SRB).
